# A Hydrophobic Ion-Paired Self-Microemulsifying System Enhances Jejunal Absorption and Acute Glycemic Control of Semaglutide in Rats

**DOI:** 10.3390/ijms27219523

**Published:** 2026-10-24

**Authors:** Ke Wang, Yijie Hu, Yicheng Jiang, Ruixin Fan, Yuzhang Bao, Sikai Wang, Qian Liang, Kaili Lu, Junjie Xue, Yi Yan, Xiaoyan Liu, Wanhui Liu, Yuanjun Zhu, Jiancheng Wang

**Affiliations:** 1 Laboratory of Innovative Formulations and Pharmaceutical Excipients, Peking University Ningbo Institute of Marine Medicine, Ningbo 315832, China; 2 Beijing Key Laboratory of Molecular Pharmaceutics and New Drug Delivery Systems, State Key Laboratory of Natural and Biomimetic Drugs, School of Pharmaceutical Sciences, Peking University, Beijing 100191, China

**Keywords:** semaglutide, jejunal absorption, oral delivery, hydrophobic ion pair, self-microemulsifying drug delivery systems, type 2 diabetes mellitus

## Abstract

Oral delivery of semaglutide offers a promising strategy for type 2 diabetes mellitus (T2DM) by improving patient adherence. However, its clinical translation is limited by low oral bioavailability, resulting from the peptide’s hydrophilicity, instability in gastrointestinal environments, and poor intestinal permeability. To address these challenges, semaglutide was converted into a hydrophobic ion-pair (HIP) complex with the cationic lipid DODAB (termed SEDAB), which was subsequently incorporated into a self-microemulsifying drug delivery system (SMEDDS), yielding SEDAB-SME. A comparative screen of two cationic amphiphiles identified DODAB as the selected counterion, which forms a stable complex with semaglutide. Ion pairing with DODAB markedly increased the lipophilicity of semaglutide, and the resulting SEDAB-SME formulation enhanced its stability in simulated intestinal fluid, effectively protecting the peptide from enzymatic degradation. In vitro, FITC-semaglutide delivered by SEDAB-SME showed markedly greater cell association than native FITC-semaglutide, and inhibitor studies suggested the involvement of a cholesterol-sensitive, raft-associated internalization route. In an intra-jejunal (i.j.) regional intestinal absorption study in SD rats, SEDAB-SME enhanced jejunal absorption of semaglutide (Frel = 0.12%) by approximately 28-fold compared with native semaglutide administered via the same i.j. route. In an acute single-dose pharmacodynamic study in STZ-induced T2DM rats, oral administration of SEDAB-SME enteric-coated capsules significantly reduced blood glucose levels. These results demonstrate that the HIP-SMEDDS strategy markedly enhanced regional jejunal absorption and produced acute glucose-lowering effects in a rodent model. This proof-of-concept study highlights a promising approach that warrants further validation of direct oral pharmacokinetics and long-term safety.

## 1. Introduction

Type 2 diabetes mellitus (T2DM) represents a globally prevalent metabolic disorder characterized by insulin resistance and the progressive decline of pancreatic β-cell function [1,2]. The GLP-1 analog semaglutide effectively addresses these defects by stimulating insulin secretion, suppressing glucagon, promoting weight loss, and demonstrating significant cardiovascular and anti-obesity benefits [3,4]. Despite the efficacy of its injectable form, oral delivery remains highly desirable to improve patient adherence. However, the oral bioavailability of therapeutic peptides such as semaglutide is extremely low, owing to enzymatic degradation, poor intestinal permeability, and the mucus barrier. The commercial oral tablet (Rybelsus^®^) employs the permeation enhancer sodium-[8-(2-hydroxybenzoyl)amino] octanoate (SNAC) but still suffers from low (0.4–1% in humans) and variable bioavailability, strict fasting requirements, fluid restrictions, and gastrointestinal side effects [5,6], highlighting the persistent challenges in oral peptide delivery [7]. Consequently, developing more efficient and robust oral delivery strategies is of paramount clinical importance to fully unlock the therapeutic potential of semaglutide.

Among various strategies, lipid-based nanocarriers like self-microemulsifying drug delivery systems (SMEDDS) are particularly promising [8]. SMEDDSs are isotropic mixtures that spontaneously form oil-in-water nanoemulsions in the gut, which encapsulate drugs to shield them from degradation and enhance mucosal penetration and transmembrane transport [9,10]. Our group’s prior work established the feasibility of this strategy, demonstrating that a tailored microemulsion could increase the oral bioavailability of an earthworm fibrinolytic enzyme by over 200-fold [11]. However, a key challenge for applying SMEDDS to semaglutide is the peptide’s inherent hydrophilicity and poor solubility in lipid excipients, which limit effective drug loading.

Hydrophobic ion pairing (HIP) addresses this solubilization hurdle by forming an electrostatic complex between the peptide and a hydrophobic counterion, neutralizing charge and enhancing lipophilicity for efficient incorporation into SMEDDSs [12,13]. The combination of HIP and SMEDDSs has been successfully explored for the oral delivery of various peptides. For instance, it has enabled the oral delivery of salmon calcitonin with significant hypocalcemic activity [14], improved the oral bioavailability of exenatide [15,16] and insulin [17] in rodents, and achieved a notable bioavailability for octreotide [18] in a porcine model. For the specific combination of HIP and SMEDDSs applied to semaglutide, an in vitro study compared HIP and reverse micelle strategies for semaglutide SMEDDSs [19]. More recently, Zhang et al. developed a HIP complex of semaglutide with sodium docusate (SET-DOC) incorporated into a self-emulsifying drug delivery system, which significantly reduced blood glucose levels and improved lipid profiles in a T2DM rat model [20]. These studies establish that the HIP-SMEDDS strategy is a viable approach for semaglutide oral delivery and that the general concept is no longer unexplored. However, semaglutide presents physicochemical challenges that differ from those of exenatide, salmon calcitonin, or octreotide: its high degree of acylation (C18 fatty diacid chain) and large molecular weight (~4.1 kDa) increase structural complexity for ion-pair formation, and its seven net negative charges at physiological pH require careful stoichiometric optimization of the cationic counterion. These semaglutide-specific considerations justify dedicated counterion screening and formulation optimization rather than direct extrapolation from other peptide systems. Indeed, the choice of counterion is particularly consequential: different pairing agents yield complexes with distinct lipophilicity, stability, and compatibility within SMEDDSs [21]. Therefore, whether an optimal HIP partner for semaglutide can be identified, whether the resulting complex is suitable for SMEDDS loading, and whether the formulation can enhance intestinal absorption and produce glycemic control in vivo have not been systematically evaluated.

To address these questions, this study aimed to develop a HIP-SMEDDS approach for oral semaglutide delivery. Through counterion screening (DODAB vs. TOAB), DODAB [22] was identified as the optimal counterion, and the resulting stable complex (SEDAB) was incorporated into a tailored SMEDDS (SEDAB-SME). We comprehensively evaluated its physicochemical properties, cellular uptake and transport mechanisms, pharmacokinetics, and acute glucoregulatory efficacy in a T2DM rat model. This work provides a thorough in vitro to in vivo validation of a DODAB-based HIP-SMEDDS approach for semaglutide, integrating mechanistic insight with jejunal absorption data and acute pharmacodynamic activity.

## 2. Results

### 2.1. Preparation and Characterization of the Semaglutide–DODAB HIP Complex (SEDAB)

**Figure 1:**
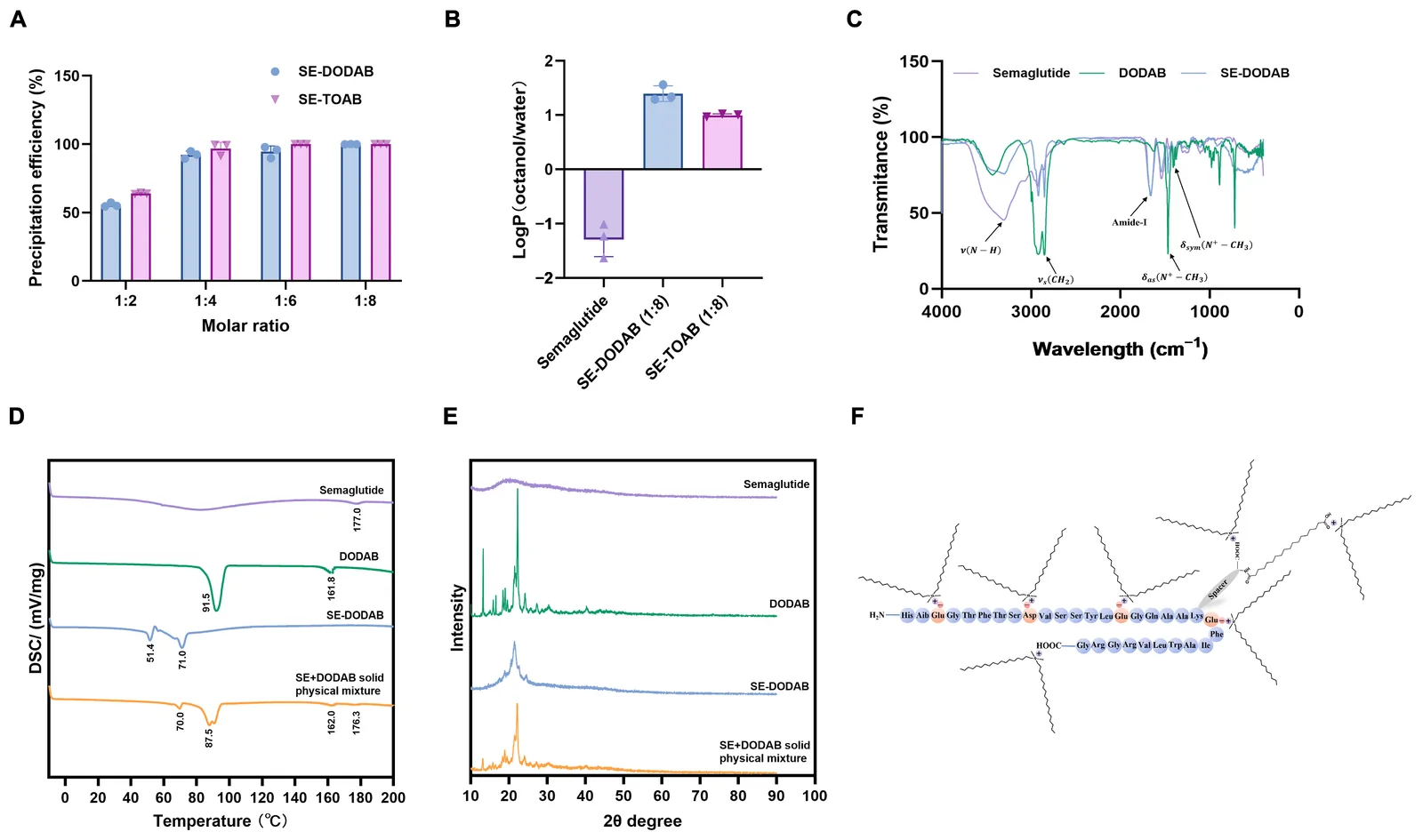
Formation and physicochemical characterization of the semaglutide–DODAB HIP complex. (**A**) Precipitation efficiency of HIP complexes formed using DODAB or TOAB as the cationic lipid at varying molar ratios (*n* = 3); (**B**) the log *P_n_*_-octanol/water_ of native semaglutide, SE-TOAB, and SE-DODAB (*n* = 3); (**C**) Fourier-transformed infrared (FTIR) spectra of native DODAB, native semaglutide, and SE-DODAB; (**D**) differential scanning calorimetry (DSC) thermograms and (**E**) X-ray powder diffraction (XRD) patterns of native semaglutide, DODAB, the semaglutide/DODAB solid physical mixture, and the semaglutide–DODAB ion-paired complex (SE-DODAB); (**F**) schematic illustration of the electrostatic interaction between semaglutide and DODAB to form the SE-DODAB complex. Data are presented as mean ± SD. For all quantitative measurements, *n* = 3 represents three technical replicates.

Hydrophobic ion pairing effectively enhances peptide lipophilicity through sub-micellar electrostatic and hydrophobic interactions [23]. Given that semaglutide carries a net negative charge (estimated to be approximately −7 at pH 7.4), two cationic lipids, TOAB and DODAB, were screened as candidate counterions, and their chemical structures are shown in Table S1. Upon ion pairing, the cationic headgroups electrostatically neutralize the anionic residues of semaglutide, while their multiple hydrocarbon chains shield the residual charges, thereby converting the hydrophilic peptide into a water-insoluble lipophilic complex that precipitates from the aqueous phase. Accordingly, the precipitation efficiency (PE%) serves as a direct readout of ion-pair complexation. Both lipids efficiently induced complexation, and complete precipitation (100%) was achieved at the highest molar ratio screened (1:8, peptide:lipid) for both counterions (Figure 1A).

The lipophilicity of the HIP complexes prepared at the optimized 1:8 molar ratio was then assessed by determining the *n*-octanol/water partition coefficient (log *p*). Native semaglutide exhibited high hydrophilicity with a log *p* of −1.29 ± 0.31. Upon complexation, the log *p* value increased markedly, reaching 1.40 ± 0.14 for SE-DODAB and 1.00 ± 0.03 for SE-TOAB (Figure 1B). This pronounced positive shift (by approximately 2.7 log units) confirms the successful conversion of semaglutide into a lipophilic complex. Based on its superior lipophilicity, the SE-DODAB complex (hereafter referred to as SEDAB) was selected and carried forward to all subsequent studies.

The electrostatic basis of complex formation was further verified by Fourier-transform infrared (FTIR) spectroscopy. In the spectrum of native DODAB, two characteristic absorption bands located at 1468 cm^−1^ and 1398 cm^−1^ correspond to the asymmetric bending vibration δas (N^+^-CH_3_) and symmetric umbrella-type bending vibration δsym (N^+^-CH_3_) of the trimethylammonium headgroup, respectively [24,25]. Compared with pristine DODAB, the SEDAB complex exhibited a noticeable reduction in the absorption intensity at 1468 cm^−1^, and the δsym (N^+^-CH_3_) band exhibited a slight red-shift from 1398 cm^−1^ to 1396 cm^−1^. These spectral perturbations demonstrate strong electrostatic interactions between the positively charged N^+^-CH_3_ groups of DODAB and negatively charged carboxylate groups of native semaglutide, indicating the fabrication of the SE-DODAB complex (Figure 1C). To provide complementary evidence for complex formation, differential scanning calorimetry (DSC) analysis was performed (Figure 1D). Native semaglutide exhibited a broad and relatively featureless thermal profile without a sharp melting endotherm, consistent with the reported amorphous nature of solid-state semaglutide [26]. Native DODAB displayed a pronounced endothermic transition at approximately 91.5 °C, in good agreement with reported transitions of highly ordered DODAB, together with a weaker high-temperature event [27]. The semaglutide/DODAB physical mixture retained this DODAB-associated event, whereas it was no longer evident in the isolated SEDAB complex, indicating substantial alteration of DODAB molecular packing following complex preparation.

XRD analysis was further performed to investigate changes in solid-state organization following complex formation (Figure 1E). Native semaglutide exhibited a broad diffuse diffraction profile without distinct sharp Bragg reflections, consistent with its predominantly amorphous solid-state characteristics [28]. In contrast, native DODAB showed multiple sharp crystalline reflections in the wide-angle region, reflecting its highly ordered crystalline organization [29,30]. The semaglutide/DODAB physical mixture retained the major DODAB-associated crystalline reflections, indicating that simple physical mixing did not substantially disrupt the crystalline packing of DODAB. In contrast, these sharp reflections were markedly attenuated or absent in the isolated semaglutide–DODAB HIP complex, which instead exhibited a predominantly broad diffuse pattern. The loss of the characteristic DODAB reflections indicates substantial disruption of its original long-range crystalline order and formation of a more disordered solid-state organization following complex preparation. The clear difference between the HIP complex and the corresponding physical mixture suggests that this structural reorganization cannot be attributed solely to simple coexistence of the two components. Together with the FTIR, DSC, and lipophilicity results, the XRD findings provide complementary physicochemical evidence supporting formation of the semaglutide–DODAB hydrophobic ion-paired complex (Figure 1C–F).

Visual observation and supernatant analysis provided additional qualitative support. Semaglutide alone remained clear, whereas the semaglutide–DODAB system became markedly turbid and formed a precipitate (Figure S1). The semaglutide concentration in the supernatant was almost undetectable after complexation, indicating that the majority of semaglutide had been incorporated into the precipitated phase.

### 2.2. Formulation Development and Characterization of SEDAB-Loaded Self-Microemulsion (SEDAB-SME)

**Figure 2:**
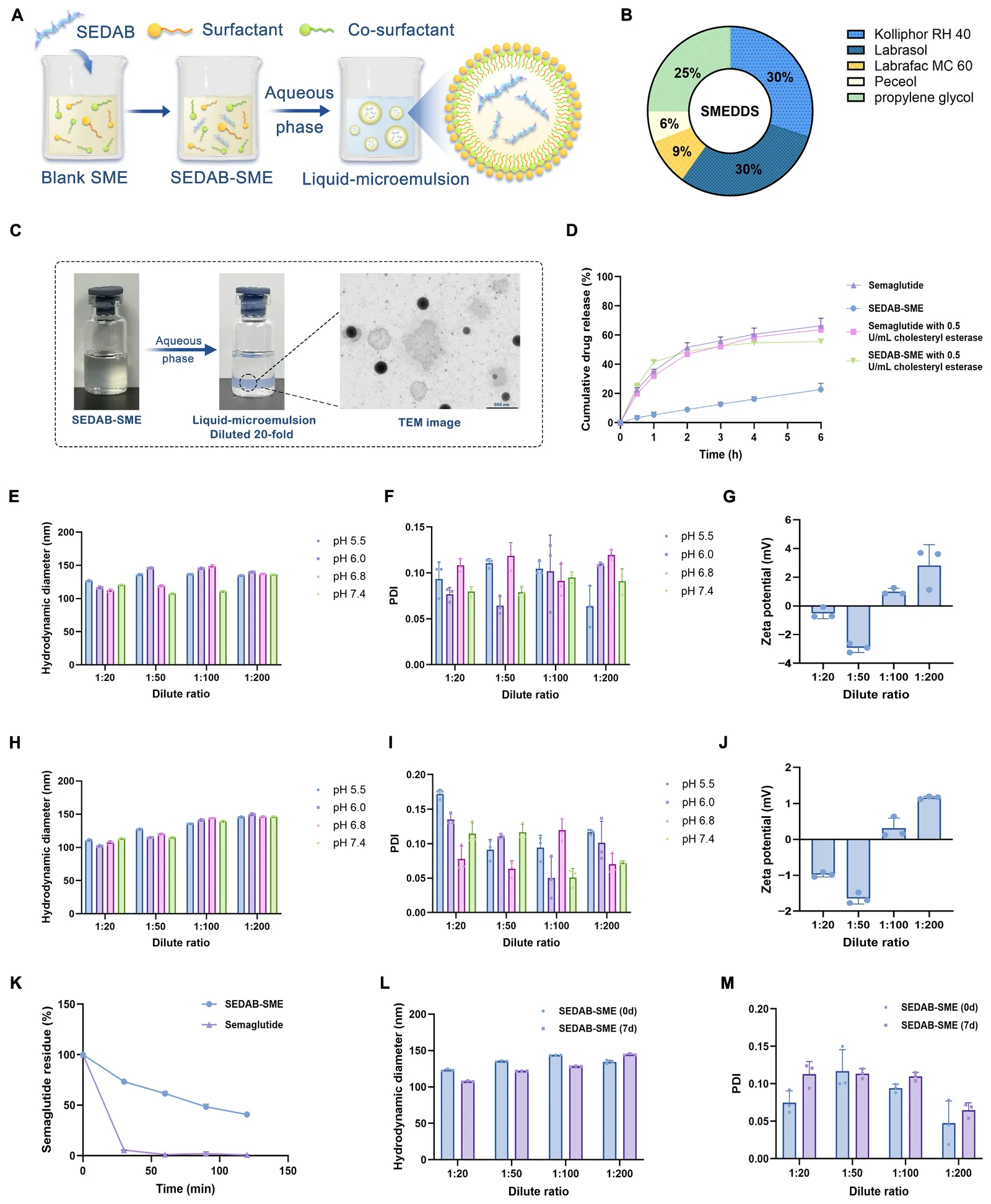
Development and in vitro evaluation of the SEDAB-loaded self-microemulsion. (**A**) Schematic diagram of the SEDAB-SME preparation process; (**B**) composition of the selected SMEDDS formulation; (**C**) visual appearance of the oil phase (left) and microemulsion phase (right), and representative TEM image of the microemulsion (scale bar = 500 nm); (**D**) in vitro cumulative drug release profiles of native semaglutide and SEDAB-SME in pH 6.8 phosphate buffer (*n* = 3). Cholesteryl esterase (0.5 U/mL) was included in both groups to provide an intestinally relevant esterase condition. The release profile of SEDAB-SME in the absence of cholesteryl esterase is also shown; (**E**–**G**) physical characterization of SEDAB-SME following dilution (1:20 to 1:200, *v*/*v*) in media at pH 5.5, 6.0, 6.8, and 7.4 at day 0: (**E**) hydrodynamic diameter (D_h_), (**F**) polydispersity index (PDI), and (**G**) zeta potential (*n* = 3); (**H**–**J**) physical characterization of SEDAB-SME after 7 days of storage at 2–8 °C and subsequent dilution: (**H**) D_h_, (**I**) PDI, and (**J**) zeta potential (*n* = 3); (**K**) proteolytic stability of native semaglutide and semaglutide incorporated in SEDAB-SME in simulated intestinal fluid (SIF); (**L**,**M**) comparison of (**L**) D_h_ and (**M**) PDI of SEDAB-SME diluted in SIF, using freshly prepared preconcentrate (day 0) or preconcentrate stored at 2–8 °C for 7 days (*n* = 3). Data are presented as mean ± SD. For all quantitative measurements, *n* = 3 represents three technical replicates.

To successfully develop a SMEDDS for SEDAB (Figure 2A), excipients were screened based on drug solubility and microemulsion-forming capability (Table 1). Among the oils screened, SEDAB exhibited exceptionally high solubility in Labrafac MC60 (6.46 ± 1.87 mg/mL). Consequently, Labrafac MC60 was selected not only for its superior solubilizing capacity but also for its reported ability to modulate cell membrane fluidity and potentially enhance cellular uptake. Additionally, Peceol, a long-chain monoolein, was incorporated into the formulation due to its potential protease inhibition activity, which is particularly beneficial for peptide stability. Labrasol ALF and Kolliphor RH 40 were selected as surfactants due to their dual function as effective emulsifiers and permeation enhancers.

As the co-surfactant, propylene glycol was selected based on its superior performance in stabilizing the microemulsion system and improving the miscibility and fluidity of the preconcentrate. The overall composition of 60:25:15 (surfactant/co-surfactant/oil, *w*/*w*) was selected with reference to reported SMEDDS phase-behavior studies, in which stable self-emulsifying regions were obtained at approximately 50–80% surfactant, 10–45% co-surfactant, and 5–15% oil depending on the excipient combination [23,31]; the oil fraction was kept at the upper end of this range to provide a sufficient lipophilic domain for the hydrophobized SEDAB complex. The formulation was subsequently screened and confirmed based on self-emulsification performance and droplet characteristics (Figure 2B). Under the selected formulation, the drug loading capacity of SEDAB was determined to be 9.5 ± 0.8 mg/mL. The semaglutide concentration in independently prepared SEDAB-SME batches ranged from approximately 8 to 10 mg/mL, as determined by HPLC (Table S5). The resulting SEDAB-loaded SMEDDS is hereafter referred to as SEDAB-SME.

Upon aqueous dilution, the SEDAB-SME preconcentrate rapidly self-emulsified into a microemulsion. TEM imaging revealed that the resulting nanodroplets were spherical and well-dispersed (Figure 2C). Furthermore, in vitro drug release profiling was performed under two conditions. In the presence of cholesteryl esterase (0.5 U/mL), which was included in both the native semaglutide and SEDAB-SME groups to assess the effect of esterase on drug release, SEDAB-SME exhibited a release profile similar to that of native semaglutide under the tested conditions (Figure 2D), with cumulative release reaching approximately 60% over 6 h. In the absence of cholesteryl esterase, SEDAB-SME released only approximately 20% of the peptide over the same period. In contrast, the release profile of native semaglutide was similar in the presence and absence of cholesteryl esterase, reaching approximately 60% cumulative release at 6 h under both conditions. These findings suggest that cholesteryl esterase may facilitate semaglutide release from SEDAB-SME, potentially through disruption of the formulation interface. The lower release observed in the absence of esterase is consistent with greater retention of semaglutide within the formulation.

Characterization of the selected SEDAB-SME across a broad dilution range (1:20 to 1:200, *v*/*v*) in media at pH 5.5, 6.0, 6.8, and 7.4 demonstrated the formation of nanodroplets with a mean hydrodynamic diameter (Dh) of approximately 100–150 nm, low PDI values, and a zeta potential within the range of −5 to 5 mV (Figure 2E–G). This near-neutral surface charge is favorable for minimizing electrostatic disruption of biological membranes, thereby potentially enhancing biocompatibility. After 7 days of storage at 2–8 °C, the measured physical parameters and self-emulsification characteristics remained within ranges similar to those observed at day 0 upon 20- to 200-fold dilution (Figure 2H–J). However, the semaglutide content decreased to 82.1% of the initial value over 7 days (Table S6), corresponding to an approximately 18% loss. The cause of this loss was not determined in the present study. These results therefore indicate only that the measured physical characteristics and self-emulsification capacity were maintained over the 7-day observation period; chemical and drug-content stability have not been established.

More importantly, SEDAB-SME provided effective protection against semaglutide degradation in simulated intestinal fluids (SIF), whereas native semaglutide underwent rapid proteolysis under identical conditions (Figure 2K). This protective effect is anticipated to minimize premature drug loss. In addition, after 7 days of storage at 2–8 °C, the SEDAB-SME preconcentrate still self-emulsified into a microemulsion with D_h_ and PDI comparable to those of freshly prepared samples upon dilution in SIF (Figure 2L,M), indicating that short-term storage did not compromise the self-emulsification capacity of the preconcentrate.

### 2.3. Enhanced Cellular Uptake and Transepithelial Transport of SEDAB-SME in Caco-2 Cells

**Figure 3:**
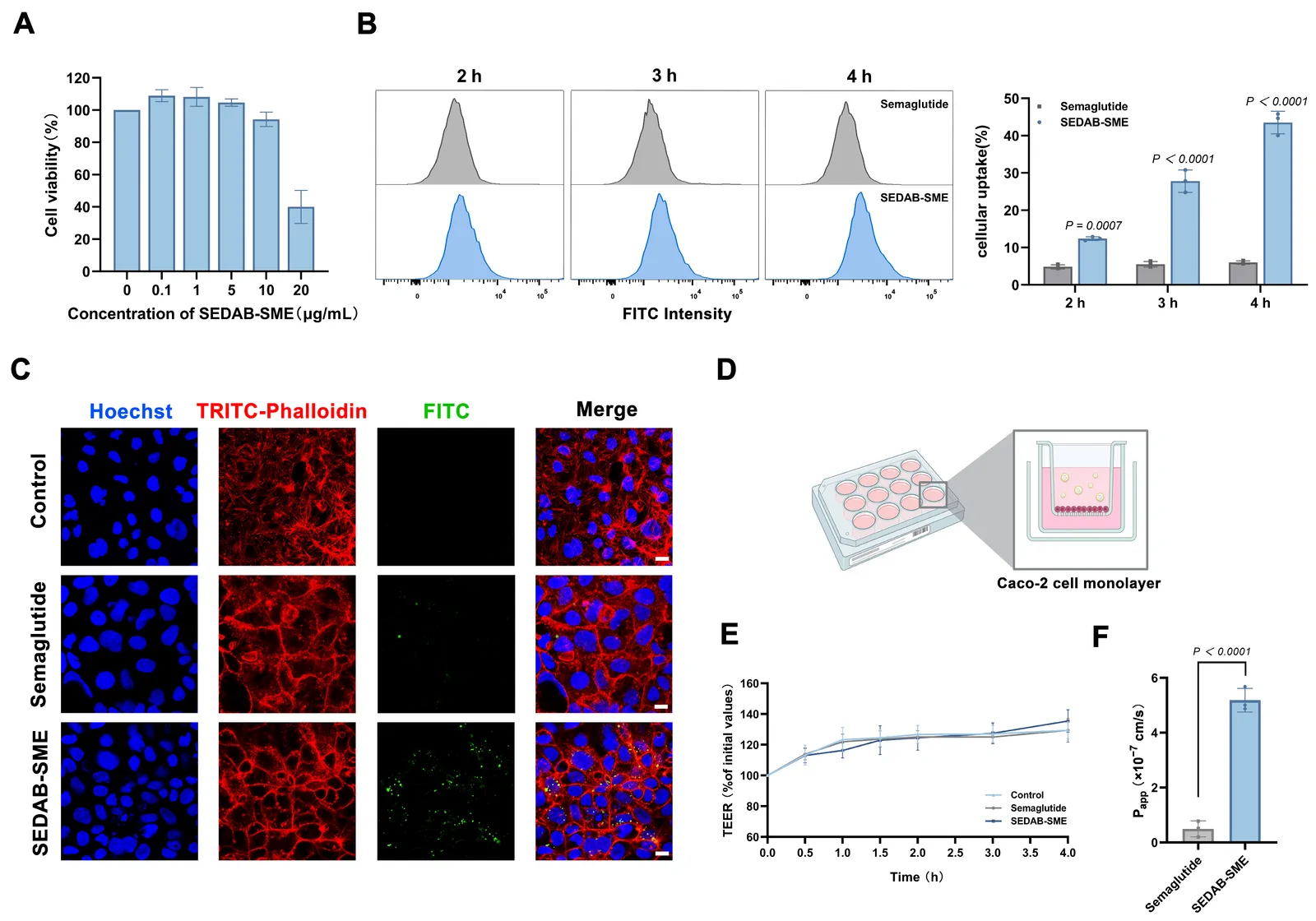
Cellular uptake and transport of SEDAB-SME in Caco-2 cells. (**A**) Cell viability of Caco-2 cells after treatment with SEDAB-SME for 5 h (*n* = 5); (**B**) quantitative analysis of cellular uptake of FITC-semaglutide and FITC-labeled SEDAB-SME by flow cytometry (*n* = 3); (**C**) representative confocal laser scanning microscopy (CLSM) images of cellular uptake. Nuclei and cytoskeleton were stained with Hoechst 33342 (blue) and TRITC-phalloidin (red), respectively. Scale bar = 20 μm. (**D**) Schematic illustration of the Caco-2 cell monolayer transwell system used for transport studies; (**E**) transepithelial electrical resistance (TEER) values across Caco-2 monolayers after treatment with HBSS, native semaglutide, or SEDAB-SME (*n* = 3); (**F**) apparent permeability coefficient (*P*app) of semaglutide across Caco-2 monolayers following SEDAB-SME treatment (*n* = 3). Data are presented as mean ± SD. Data in panel B were analyzed using two-way ANOVA followed by Sidak’s test, and data in panel F were analyzed using an unpaired two-tailed Student’s *t*-test. For all quantitative measurements, *n* = 3 and *n* = 5 denote three and five biological replicates, respectively.

Prior to conducting cellular studies, the cytotoxicity of SEDAB-SME was assessed using a CCK-8 assay in Caco-2 cells. As shown in Figure 3A, SEDAB-SME exhibited negligible cytotoxicity at concentrations up to 10 μg/mL over a 5-h incubation period. Therefore, a concentration of 10 μg/mL was selected for all subsequent experiments to ensure cell viability. To evaluate internalization efficiency, Caco-2 cells were incubated with FITC-labeled formulations. Flow cytometry analysis revealed that, for FITC-labeled SEDAB-SME, the percentage of FITC-positive cells reached 43.53 ± 3.02% after 4 h (Figure 3B), which was significantly higher than that of native FITC-semaglutide. Furthermore, the fluorescence intensity in the SEDAB-SME group exhibited a time-dependent increase, indicating progressive cellular accumulation. These quantitative findings were visually corroborated by CLSM, which displayed intense intracellular green fluorescence in the SEDAB-SME group, whereas only negligible fluorescence was observed in the native semaglutide group (Figure 3C). Because cells were extensively washed and trypsinized prior to analysis to remove free and surface-bound fluorescence, the reported signals predominantly reflect internalized peptide. Nevertheless, quantitative surface-quenching controls were not performed, and the uptake data should be interpreted as total cell-associated fluorescence, which does not by itself distinguish intact peptide from labeled degradation products.

To further elucidate the transport mechanism across the intestinal epithelium, a Caco-2 monolayer transwell model was employed (Figure 3D). TEER measurements indicated that SEDAB-SME did not disrupt tight junctions or compromise monolayer integrity, as values remained comparable to those of the control and native semaglutide groups (Figure 3E). SEDAB-SME markedly enhanced the transepithelial permeability of semaglutide, showing a 10.5-fold higher *P*_app_ value than native semaglutide (Figure 3F). Consistent with this, the amount of semaglutide associated with the Caco-2 monolayer after the 4-h transport period was also higher in the SEDAB-SME group, indicating enhanced cellular accumulation. Taken together, the integrated SEDAB-SME formulation substantially enhanced the transepithelial transport of semaglutide without compromising monolayer integrity. While the excipient-free native semaglutide showed markedly lower transport, the relative contributions of the HIP complex (SEDAB) and the lipid excipients to this enhancement could not be quantitatively separated in the present study.

### 2.4. Uptake Pathways and Intracellular Trafficking of Semaglutide Delivered by SEDAB-SME in Caco-2 Cells

**Figure 4:**
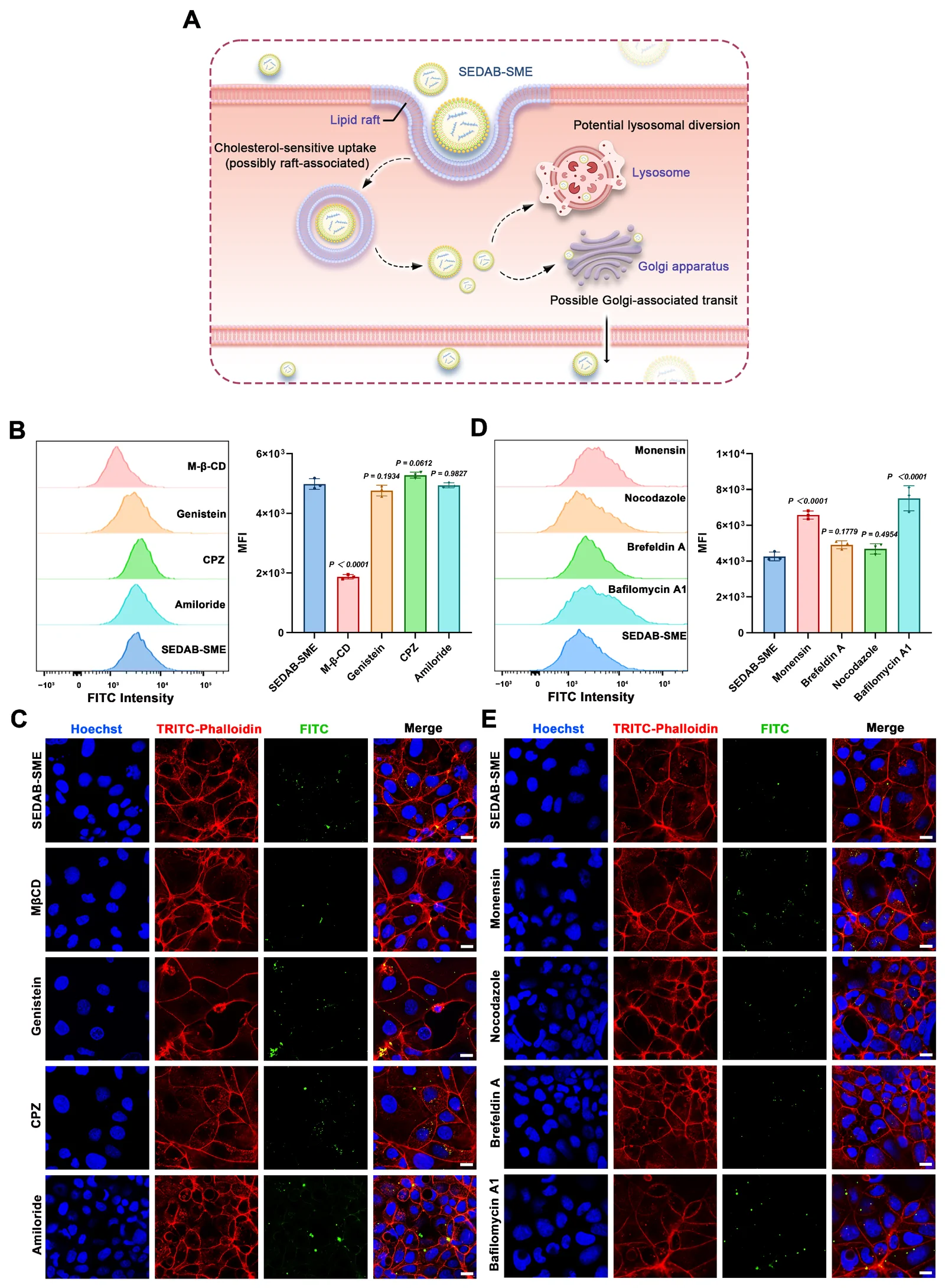
Hypothetical uptake and intracellular trafficking routes of FITC-semaglutide delivered by SEDAB-SME. (**A**) Schematic illustration of the proposed (hypothetical) pathways. All trafficking arrows are drawn as dashed lines to indicate that these are putative routes derived from indirect pharmacological inhibitor data. The fluorescence signal tracks the labeled peptide rather than the intact carrier droplet. Furthermore, the pH sensitivity of FITC and the off-target effects of the pharmacological inhibitors should be considered when interpreting these pathways. Definitive assignment of specific endocytic and intracellular trafficking pathways requires further complementary genetic or label-free approaches; (**B**) Flow cytometric analysis of the specific endocytosis inhibitors on the cellular uptake of FITC-labeled SEDAB-SME (left: representative profiles; right: quantitative analysis, *n* = 3); (**C**) Representative CLSM images of cells treated with the same panel of endocytosis inhibitors as in (**B**). In the merged images, the yellow color indicates the colocalization of FITC-semaglutide (green) and TRITC-phalloidin (red). Scale bar = 20 μm; (**D**) Flow cytometric analysis of the effects of exocytosis inhibitors on the post-internalization trafficking of FITC-labeled SEDAB-SME (left: representative profiles; right: quantitative analysis, *n* = 3); (**E**) Representative CLSM images of cells treated with the same panel of exocytosis inhibitors as in (**D**). Nuclei and cytoskeleton were stained with Hoechst 33342 (blue) and TRITC-phalloidin (red), respectively. Scale bar = 20 μm. Data are presented as mean ± SD. Data in panels B and D were analyzed using one-way ANOVA followed by Dunnett’s test. For all quantitative measurements, *n* = 3 represents three biological replicates.

To probe the endocytic routes potentially involved in the cellular association of FITC-semaglutide delivered by SEDAB-SME, four endocytosis pathway-specific inhibitors were employed: methyl-β-cyclodextrin (MβCD) and genistein for caveolae/lipid raft-mediated endocytosis, chlorpromazine (CPZ) for clathrin-mediated endocytosis, and amiloride for micropinocytosis. Flow cytometry and CLSM analyses revealed that MβCD caused the most significant reduction in cell-associated fluorescence, with an inhibition rate of 62.4%, whereas genistein did not produce a comparable reduction (Figure 4B,C). In contrast, chlorpromazine and amiloride exerted only minor effects on the uptake of FITC-labeled SEDAB-SME. Overall, the inhibitor data suggest that a cholesterol-sensitive pathway is predominantly involved in the cell association of FITC-semaglutide delivered by SEDAB-SME, consistent with, but not definitively proving, a non-canonical raft-associated route. Pharmacological inhibitors have inherent off-target effects, and complementary genetic or label-free approaches would be required for definitive pathway assignment.

To explore the post-internalization fate of the delivered peptide, we investigated its intracellular trafficking and exocytosis using inhibitors targeting specific organelles: monensin (disrupts Golgi-to-membrane transport) [32], nocodazole (inhibits microtubule-dependent vesicular movement) [33], brefeldin A (blocks ER-to-Golgi transport) [34], and bafilomycin A1 (inhibits endosome maturation into lysosomes) [35]. As illustrated in Figure 4D,E, treatment with monensin led to significant intracellular accumulation of FITC-labeled SEDAB-SME, with a 1.54-fold increase in mean fluorescence intensity (MFI) compared with the untreated control, suggesting that a fraction of the cell-associated fluorescence may transit through the Golgi apparatus en route to exocytosis. Concurrently, bafilomycin A1 treatment caused an even more substantial accumulation (1.76-fold increase in MFI), suggesting that a fraction of the internalized peptide may be diverted to lysosomes for degradation. In contrast, brefeldin A and nocodazole did not produce significant changes in intracellular SEDAB-SME levels. Overall, the inhibitor data indicate that a cholesterol-sensitive component is involved in the cell association of FITC-semaglutide delivered by SEDAB-SME. Although this is consistent with a non-canonical raft-associated route, the pharmacological nature of the inhibitors precludes definitive pathway assignment. Furthermore, while monensin and bafilomycin A1 altered intracellular fluorescence, both agents disrupt organelle pH and FITC is highly pH-sensitive; thus, these changes may partly reflect pH-dependent dequenching rather than genuine changes in intracellular trafficking. These findings are indicative of, but do not definitively prove, potential Golgi-associated transit or lysosomal diversion.

### 2.5. In Vivo Pharmacokinetics and Pharmacodynamics of SEDAB-SME in Rats

**Figure 5:**
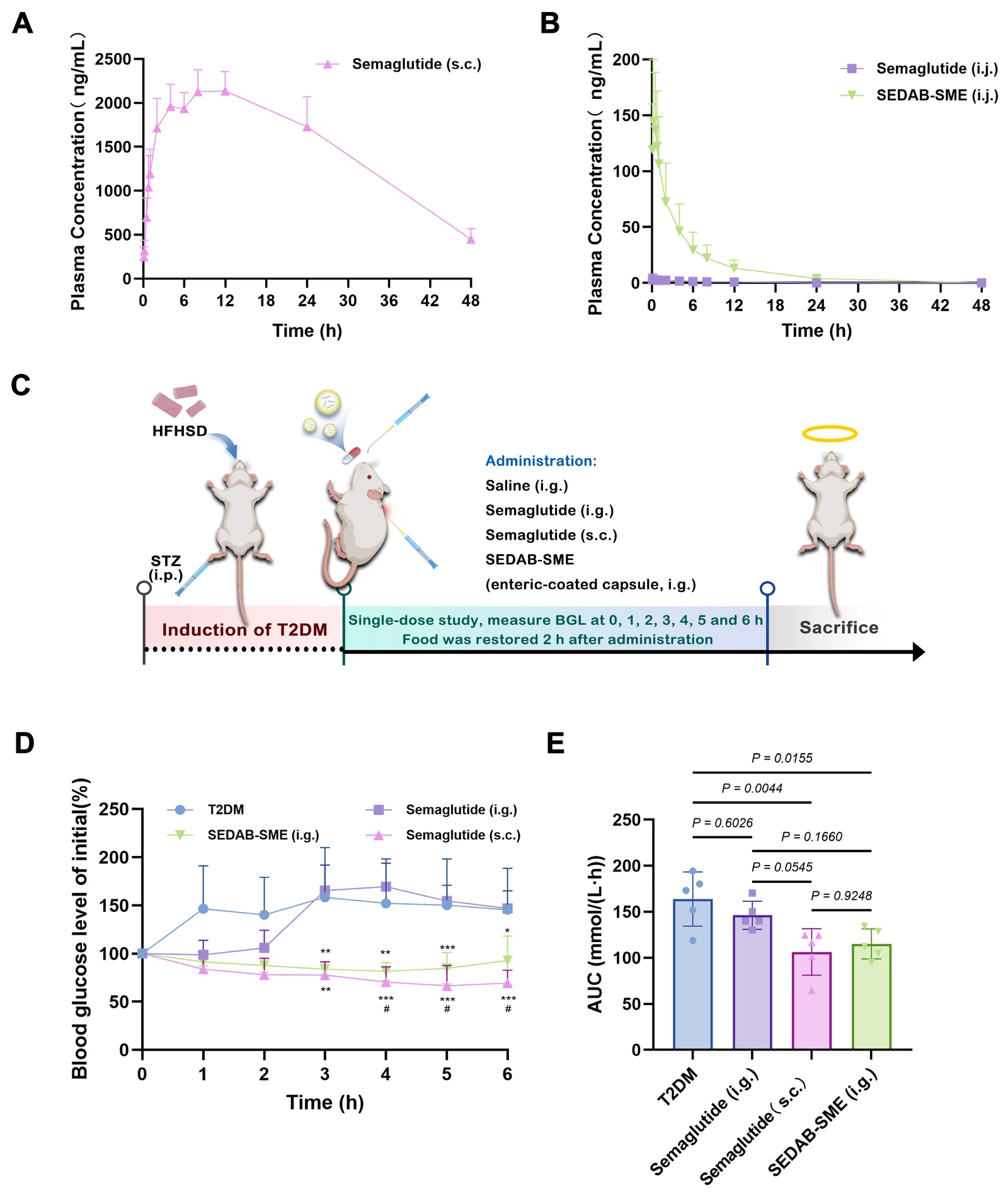
Enhanced intestinal absorption and oral hypoglycemic efficacy of SEDAB-SME. (**A**) Plasma concentration–time profiles of semaglutide following subcutaneous (s.c.) injection (reference group, *n* = 5); (**B**) comparative plasma concentration–time profiles of native semaglutide and SEDAB-SME following intra-jejunal (i.j.) administration (*n* = 5); (**C**) schematic timeline of the pharmacodynamic study design in streptozotocin (STZ)-induced T2DM rats. After model establishment, rats received a single dose of one of the following treatments: oral normal saline (T2DM control), subcutaneous (s.c.) semaglutide (0.02 mg/kg), oral native semaglutide (2 mg/kg, i.g.), and oral SEDAB-SME enteric capsules (2 mg/kg semaglutide equivalent); food was restored 2 h post-administration. (**D**) Blood glucose levels (expressed as % of initial value) over time following administration (*n* = 5); (**E**) area under the blood glucose curve (*AUC*_0–6h_) values for the different groups. Data are presented as mean ± SD. Data in panel D were analyzed using a two-way repeated-measures ANOVA (group × time) with the Geisser–Greenhouse correction. Following a significant interaction, all pairwise between-group comparisons were performed within each time point using Tukey’s multiple-comparisons test; each time point was treated as a separate family, and multiplicity-adjusted *p* values are shown. The *p* values annotated in panel D are from this model (* *p* < 0.05, ** *p* < 0.01, *** *p* < 0.001 vs. semaglutide (i.g.) group, # *p* < 0.05 vs. T2DM group). *AUC* data in panel E were analyzed using one-way ANOVA followed by Tukey’s test. For all quantitative measurements, *n* = 5 represents five biological replicates.

To quantitatively assess the intestinal absorption enhancement conferred by SEDAB-SME, we first performed a regional jejunal absorption PK study in healthy SD rats using the intra-jejunal (i.j.) route as a validated regional absorption model [36,37,38,39]. As shown in Table 2 and Figure 5A,B, SEDAB-SME administered i.j. achieved a relative bioavailability (Frel%) of 0.12% relative to subcutaneous injection, representing an approximately 28-fold increase in systemic exposure compared with native semaglutide administered via the same i.j. route (Frel = 0.004%). Notably, native semaglutide exhibited a similarly early Tmax in the i.j. model (median 0.167 h, Table 2), indicating that rapid initial appearance is a feature of the regional delivery route rather than a unique property of SEDAB-SME. Because the plasma concentrations of the native i.j. group were close to the LLOQ over much of the sampling window (Table 2), this fold-change should be interpreted as an order-of-magnitude estimate rather than a precise point value. This enhancement is consistent with the ability of the HIP-SMEDDS strategy to facilitate intestinal uptake of semaglutide.

Building on the regional intestinal absorption data, we next evaluated the acute glucoregulatory efficacy of SEDAB-SME in an STZ-induced T2DM rat model following oral administration in enteric-coated capsules (experimental timeline shown in Figure 5C). For the pharmacodynamic study, a lower therapeutic dose (2 mg/kg) was used, which was suitable for encapsulation in enteric-coated capsules. A 6-h monitoring window with food restored at 2 h post-administration was adopted, allowing simultaneous evaluation of fasting-phase (0–2 h) and postprandial (2–6 h) glycemic control. In the fasting phase (0–2 h), SEDAB-SME reduced blood glucose levels compared with the T2DM control. In the postprandial phase (2–6 h), the T2DM control and oral native semaglutide groups exhibited pronounced postprandial glucose excursions driven by food intake (Figure 5D). SEDAB-SME maintained postprandial glycemic control, and its *AUC*_0–6h_ was not significantly different from that of subcutaneous semaglutide (*p* = 0.9248, Figure 5E). Nevertheless, the subcutaneous group showed numerically lower blood glucose levels at individual time points during this phase (Figure 5D), and the subcutaneous group treatment likely acted near the plateau of the acute glucose-lowering response in this model. Therefore, therapeutic equivalence should not be inferred. Overall, oral SEDAB-SME treatment reduced the blood glucose *AUC*_0–6h_ by 29.7% compared with the T2DM control, while subcutaneous semaglutide showed a more pronounced *AUC* reduction of 35.1% at a 100-fold lower dose.

## 3. Discussion

The aim of this study was to determine whether hydrophobic ion pairing combined with a self-microemulsifying drug delivery system could overcome the poor lipophilicity of semaglutide and its susceptibility to proteolytic degradation in the gastrointestinal tract. Conversion of semaglutide into the DODAB ion-paired complex raised the *n*-octanol/water partition coefficient. Incorporation of this complex into a tailored SMEDDS enhanced jejunal absorption by approximately 28-fold and produced significant acute glycemic control in T2DM rats after oral administration, providing a depth of quantitative absorption characterization not previously available for semaglutide SMEDDSs.

The selection of the counterion was a decisive step. Previous SMEDDS designs for semaglutide based on hydrophobic ion pairs or reverse micelles were evaluated primarily at the in vitro stage [19], and a subsequent HIP-based SMEDDS study for semaglutide utilized sodium docusate as the counterion [20]. The present study extends this approach by employing DODAB, a double C18-chain quaternary ammonium lipid structurally distinct from these previously explored counterions. In the present screen, both DODAB and TOAB induced complete precipitation at a peptide to lipid molar ratio of 1:8. A modest excess of DODAB over the approximately seven net negative charges of semaglutide was used to promote sufficiently complete association [23]. This ratio should be regarded as a nominal molar ratio used for preparation, rather than as evidence that one semaglutide molecule forms a discrete complex with exactly eight DODAB molecules. The exact stoichiometry of the complex was not determined in this study. This is consistent with other HIP-based systems, such as the semaglutide–sodium docusate system reported by Zhang et al. [20], where a ratio at or slightly above charge neutrality was used. Lipophilicity ultimately distinguished the two candidates, as the longer C18 alkyl chains of DODAB yielded a higher partition coefficient than the C8 chains of TOAB (1.40 ± 0.14 versus 1.00 ± 0.03). Fourier-transform infrared spectroscopy supported the electrostatic mechanism through the reduced intensity and red shift of the N^+^-CH_3_ bending bands [24,25]. Notably, SEDAB itself remained a water-insoluble precipitate, a property that precludes direct oral administration yet is required for efficient partitioning into a lipid vehicle, and this motivated the design of SEDAB-SME.

The two technologies proved complementary rather than additive. Ion pairing supplied the lipophilicity needed for drug loading, as reflected by the high solubility of SEDAB in Labrafac MC60 (6.5 ± 1.9 mg/mL) and the loading capacity of 9.5 ± 0.8 mg/mL in the selected formulation. The SMEDDS supplied the functions that the complex lacks. Upon dilution, the preconcentrate self-emulsified into nanodroplets with a Dh of approximately 100 to 150 nm with a low PDI and a near neutral zeta potential, and these measured physical characteristics remained within similar ranges across dilution ratios, pH values from 5.5 to 7.4, and after 7 days of storage at 2–8 °C. However, given the observed approximately 18% loss of semaglutide content over 7 days, no claims regarding drug-content, chemical or long-term storage stability can be made, and further optimization to prevent peptide loss is required. This pH window reflects the dosage-form design: the formulation is administered in HPMCP enteric-coated capsules dissolving above pH approximately 5.5 and is thus never exposed to gastric fluid. The acute glucose lowering after oral dosing (Figure 5D,E) indicates that the formulation reaches the intestine in its protected state. Functionally, SEDAB-SME markedly retarded proteolysis in simulated intestinal fluid, whereas native semaglutide degraded rapidly under identical conditions. In the release assay, SEDAB-SME released only approximately 20% of the peptide over 6 h in the absence of cholesteryl esterase, which is consistent with greater retention of semaglutide within the formulation. In the presence of cholesteryl esterase, SEDAB-SME showed an increased semaglutide release profile that approached that of native semaglutide under the conditions of the in vitro release assay. Cholesteryl esterase was selected as an intestinally relevant esterase to examine whether esterase-mediated disruption of the formulation interface could facilitate peptide release. This esterase-facilitated release may be attributed to the large interfacial area of the self-emulsified microemulsion droplets, which allows for rapid partitioning of the released peptide into the aqueous phase upon disruption of the interfacial film. The observed increase in release is consistent with such an effect. However, this experiment does not establish complete dissociation of the formulation, the fraction of intact semaglutide released in vivo, or its subsequent availability for intestinal absorption. Therefore, the release data are interpreted only as supporting cholesteryl esterase-facilitated peptide release under the tested in vitro conditions.

At the cellular level, the integrated SEDAB-SME formulation enhanced transepithelial transport across Caco-2 monolayers by 10.5-fold without compromising monolayer integrity. The enhancement is most consistent with a predominantly transcellular mechanism, since the surfactant concentrations reaching the monolayer (0.03% *w*/*v*) were far below the thresholds reported to open paracellular tight junctions [40,41]. It should be noted that a blank-vehicle control (blank SME) was not included in this study. Since Labrasol is itself a documented permeation enhancer [40,41], a contribution of the excipients to the observed enhancement through mechanisms other than tight junction opening (e.g., membrane fluidization) cannot be excluded. Therefore, the observed permeability enhancement is attributed to the integrated SEDAB-SME formulation rather than the HIP complex alone. TEER values were preserved, and previous SMEDDS studies reported intact epithelial barriers under comparable conditions [9]. However, TEER alone cannot definitively exclude transient paracellular contributions, and the rapid in vivo appearance of semaglutide (Tmax ≈ 10 min, Table 2) may additionally reflect a paracellular or pericellular component alongside the slower transcellular pathway characterized in vitro; the two routes are not mutually exclusive, and the absorption enhancement conferred by SEDAB-SME is reflected primarily in the magnitude of systemic exposure (AUC) rather than in the absorption onset rate. Future studies employing paracellular markers (e.g., Lucifer Yellow) and mass-balance recovery analysis, and a blank SME control would help quantify the relative contributions of each pathway and the excipients.

The inhibitor data indicate that the cell association of FITC-semaglutide delivered by SEDAB-SME involves a cholesterol-sensitive component, potentially via a raft-associated route; MβCD caused the strongest inhibition of uptake (62.4%), whereas genistein, which blocks the Src-caveolin-1 step of classical caveolae budding, did not, which may be consistent with the involvement of non-canonical raft routes such as CLIC/GEEC or flotillin-dependent uptake [42,43,44,45,46]. The weak effects of chlorpromazine and amiloride argue against major contributions of clathrin-mediated endocytosis and macropinocytosis. However, these pharmacological assignments remain indicative rather than definitive, because such inhibitors possess off-target effects; for example, MβCD alters membrane properties beyond cholesterol depletion. After internalization, monensin increased intracellular fluorescence by 1.54-fold, which may reflect Golgi transit before exocytosis [32]. Bafilomycin A1 produced an even stronger accumulation (1.76-fold), potentially indicating lysosomal diversion as a possible competing fate. Importantly, both agents alter endosomal and Golgi pH, and because FITC fluorescence is highly pH-sensitive, these apparent accumulations may partly reflect pH-dependent dequenching rather than a true blockade of intracellular transit. Brefeldin A and nocodazole had no significant effect, arguing against major involvement of the retrograde ER-to-Golgi route and microtubule-dependent transport [33,34]. Compared with the baicalein-loaded SMEDDS, which distributed broadly across endolysosomal and ER/Golgi compartments [47], SEDAB-SME appeared to favor a cholesterol-sensitive route; however, a definitive raft-to-Golgi routing cannot be established from these indirect data. The lysosomal bottleneck nevertheless represents a rational target, and the incorporation of pH-responsive or endosomolytic excipients may redirect this fraction and further enhance intestinal absorption.

In the regional jejunal absorption model, SEDAB-SME increased the relative bioavailability from 0.004% to 0.12% relative to subcutaneous injection, corresponding to an approximately 28-fold enhancement of systemic exposure. In addition, the i.j. model involves a surgical cannulation procedure; although animals recovered for at least 96 h before dosing, a residual effect of the procedure on absolute absorption cannot be excluded, and this limitation applies equally to both i.j. groups of the present comparison. Although direct oral PK was not measured, oral administration of enteric-coated capsules was associated with acute pharmacodynamic activity in STZ-induced T2DM rats, where fasting-phase glucose lowering was followed by suppression of the pronounced postprandial excursions seen in the control groups, reducing the *AUC*_0–6h_ by 29.7%, whereas subcutaneous semaglutide achieved 35.1% at a 100-fold lower dose. This two-phase pattern is consistent with the clinical pharmacology of GLP-1 receptor agonists [48,49] and with a previous report of oral semaglutide efficacy in STZ-induced diabetic rats [50]. The magnitude of enhancement is in line with reported hydrophobic ion-paired SMEDDSs of insulin, exenatide and octreotide [14,15,16,17,18,21]. Although the acute design does not permit a quantitative PK/PD linkage, the glucose-lowering response increased in the same order as the estimated systemic exposure across the four groups (saline control ≈ oral native semaglutide < oral SEDAB-SME < subcutaneous semaglutide; Figure 5D,E). Based on the intra-jejunal PK data, the estimated systemic exposure after oral SEDAB-SME (2 mg/kg, Frel = 0.12%) was approximately 12% of that after subcutaneous semaglutide (0.02 mg/kg). The subcutaneous comparator thus achieved an approximately 8-fold higher estimated exposure. The difference in glucose lowering was modest: 35.1% *AUC*_0–6h_ reduction for subcutaneous semaglutide versus 29.7% for oral SEDAB-SME. Because direct oral pharmacokinetics were absent and the routes/doses differed, the similar glucose profiles must not be interpreted as evidence of oral bioavailability enhancement or therapeutic equivalence. Despite this exposure difference, this pattern is most consistent with the 0.02 mg/kg subcutaneous dose lying beyond the steep portion of the glucose-lowering dose–response curve. This interpretation is supported by two lines of evidence. First, the glucose-dependent pharmacology of GLP-1 receptor agonists imposes a physiological ceiling on the maximum achievable glucose reduction. Second, a saturating exposure–response relationship has been reported for semaglutide in clinical studies [51]. In addition, GLP-1 receptor agonists can produce clinical effects at low receptor occupancy (approximately 1–2%) [52]. Their pharmacodynamic response is therefore saturable. In the initial preclinical characterization of semaglutide, the glucose-lowering dose–response of subcutaneous semaglutide in db/db mice exhibited an EC50 of <2 nmol/kg, and the profiles at 10, 30, and 100 nmol/kg were largely superimposable, indicating that the response approached its plateau at approximately 10 nmol/kg, which corresponds to approximately 0.02 mg/kg in rats under standard mouse-to-rat translation (Km-based, 1:0.5) and thus matches the comparator dose used here [53]. In a target-mediated drug disposition model of liraglutide in diabetic rats, the blood glucose reduction exhibited a saturating relationship with both dose and plasma exposure [54]. In this model, receptor occupancy was a more relevant determinant of the pharmacodynamic response than plasma drug concentration. These observations indicate that the subcutaneous treatment likely acted near the plateau of the acute glucose-lowering response in this model. The similar glucose profiles should therefore not be interpreted as indicating equivalent systemic exposure or therapeutic equivalence. A dedicated positive-control group (e.g., semaglutide co-administered with SNAC, the clinical benchmark) was not included in the present study and represents a rational reference for future benchmarking. The absolute absorbed fraction remains low, however, as expected for a large acylated peptide and given that the oral bioavailability of the marketed tablet in humans is only 0.4 to 1% [5,6].

A further consideration concerns the fate and safety of DODAB. Upon intestinal delivery, the SEDAB complex resides within the microemulsion droplets, but exposure to bile salts and endogenous lipids may promote partial dissociation of the ion pair and release of free DODAB. As a cationic lipid, DODAB possesses well-documented membrane-disruptive activity [55,56,57]. and would be expected to interact strongly with the negatively charged mucus layer, potentially aiding retention near the absorptive site yet also perturbing the mucus barrier upon repeated exposure. Importantly, in vivo studies of DODAB-containing formulations, albeit at high carrier doses via parenteral routes, have identified the cationic carrier as a contributor to hepatic and splenic toxicity signals, which the authors attributed primarily to the route of administration [58,59]. These findings were obtained at approximately 30-fold higher doses under repeated parenteral dosing and cannot be extrapolated directly to the single-dose oral microemulsion presented here, in which systemic exposure to DODAB is expected to be minimal. Nevertheless, they underscore that the repeated-dose oral toxicity, mucosal compatibility, and long-term safety of DODAB as a pharmaceutical excipient remain to be systematically established. In the present study, safety assessment was accordingly limited to an in vitro CCK-8 assay in Caco-2 cells (0.1–20 μg/mL, 5 h), with negligible cytotoxicity up to 10 μg/mL. The present system should therefore be regarded as a proof-of-concept formulation rather than a clinically translatable product at this stage.

Several limitations should be acknowledged. First, the quantitative absorption data were obtained via intra-jejunal administration rather than oral dosing; the enhancement therefore reflects jejunal absorption rather than directly measured oral bioavailability, and the fraction absorbed after gastric transit could not be quantified. The relative bioavailability versus subcutaneous injection was very low (Frel = 0.12%); the absolute oral bioavailability was not measured, and its clinical significance remains to be established. Second, the stability assessment was limited to a 7-day refrigerated period at 2–8 °C and did not include accelerated or long-term storage conditions. Although the measured physical characteristics and self-emulsification behavior remained largely unchanged over this period, semaglutide content decreased to 82.1% of the initial value, corresponding to an approximately 18% loss. The cause of this loss remains unresolved because degradation-product analysis, mass-balance recovery, and other studies capable of distinguishing chemical degradation from processes such as adsorption or precipitation were not performed. Therefore, the present data do not establish drug-content or chemical stability, and no conclusions regarding long-term storage stability can be drawn. Further formulation optimization, dedicated degradation-product analyses, mass-balance studies, and longer-term stability studies are required. Third, only a single acute dose was tested, and safety was limited to an in vitro Caco-2 assay; chronic multidose efficacy, intestinal mucosal integrity, repeated-dose toxicity, mucus interaction, and the long-term oral safety of DODAB remain to be established. Fourth, the mechanistic evidence is indirect: the FITC label tracks the peptide rather than the carrier droplet, and pH-sensitive fluorescence, inhibitor off-target effects, and the absence of surface-quenching and paracellular-marker controls mean that the pathway assignments are indicative rather than definitive. Moreover, because no blank-vehicle (blank SME) control was included in the transport experiments, the relative contributions of the SEDAB complex and the excipients to the observed permeability enhancement could not be separated; the enhancement is accordingly attributed to the integrated formulation, and blank-vehicle controls are identified as required in future studies. The Caco-2 model lacks the mucus layer, so the uptake data represent an upper estimate. Fifth, the PK and PD studies employed different routes, doses, and animal models, precluding a direct quantitative linkage between systemic exposure and glycemic effect; the acute single-dose design with a 6-h monitoring window does not assess sustained glycemic control. With *n* = 5 and the inherent variability of STZ-induced hyperglycemia, the findings support proof-of-concept rather than therapeutic equivalence; dedicated control groups (a blank enteric-coated capsule group and a positive control such as semaglutide co-administered with SNAC) were also not included, precluding benchmarking against the clinical standard.

Taken together, and notwithstanding the limitations outlined above, this study established a mechanistically characterized hydrophobic ion-paired SMEDDS approach for enhancing the intestinal absorption of semaglutide. Hydrophobic ion pairing with DODAB converted the hydrophilic peptide into a lipophilic complex suitable for efficient lipid loading, and the SMEDDS vehicle provided enzymatic protection in simulated intestinal fluid. Intra-jejunal administration of the selected formulation enhanced semaglutide jejunal absorption approximately 28-fold relative to the native peptide (Frel = 0.12% vs. subcutaneous injection), and oral administration of enteric-coated capsules achieved acute glucose lowering in STZ-induced T2DM rats. However, these findings are strictly limited to the demonstrated enhancement of regional jejunal absorption and the acute pharmacodynamic response; the data do not establish oral bioavailability enhancement or therapeutic equivalence. These results should be interpreted in light of the limitations discussed above, including the intra-jejunal rather than oral pharmacokinetic assessment, the short-term stability evaluation, the acute single-dose design, and the absence of long-term oral safety data for DODAB. Beyond semaglutide, the present findings provide preliminary evidence that this strategy may be applicable to other hydrophilic macromolecular therapeutics, although further validation of oral pharmacokinetics, comprehensive safety evaluation, and demonstration of generality across other peptides remain necessary before its translational potential can be realized.

## 4. Materials and Methods

### 4.1. Materials

Semaglutide was obtained from JYMed Technology Co., Ltd. (Shenzhen, China). Lipid and surfactant excipients, including Labrafac™ MC60, Capryol^®^ 90, Transcutol^®^ P, Labrasol^®^ ALF and Peceol^®^ were provided by Gattefossé (Saint-Priest, France). Kolliphor^®^ HS 15 and Kolliphor^®^ RH 40 were supplied by BASF (Ludwigshafen, Germany), propylene glycol, ethanol, and Tween 80 were obtained from Macklin Biochemical Co., Ltd. (Shanghai, China). MCT, PEG 400 and cholesteryl esterase were purchased from Yuanye Bio-Technology Co., Ltd. (Shanghai, China). Cationic lipids dioctadecyldimethylammonium bromide (DODAB) and tetraoctylammonium bromide (TOAB) were also purchased from Yuanye Biotechnology Co., Ltd. (Shanghai, China). Cell culture media (MEM, DMEM) were sourced from Pricella Biotechnology Co., Ltd. (Wuhan, China); fetal bovine serum was obtained from ExCell Bio Technology Co., Ltd. (Suzhou, China) and trypsin was purchased from Thermo Fisher Scientific (Waltham, MA, USA); and Transwell^®^ inserts were purchased from Corning (Corning, NY, USA). Biochemical reagents and assay kits, including penicillin–streptomycin, HBSS, PBS, Hoechst 33342, paraformaldehyde, monensin, nocodazole and cell counting kit-8 were acquired from Beyotime Biotechnology (Shanghai, China). Tetramethylrhodamine (TRITC)-phalloidin was provided by Yeasen Biotechnology Co., Ltd. (Shanghai, China). Pathway inhibitors (MβCD, chlorpromazine, genistein, amiloride, brefeldin A, and bafilomycin A1) were purchased from Macklin Inc. (Shanghai, China). All other organic reagents were of analytical grade and obtained from Sigma-Aldrich (St. Louis, MI, USA).

### 4.2. Cells and Animals

The Caco-2 human colorectal adenocarcinoma cell line was purchased from Pricella Biotechnology Co., Ltd. (Wuhan, China). Caco-2 cells were cultured in Minimum Essential Medium (MEM) supplemented with 10% fetal bovine serum (FBS), 1% non-essential amino acid (NEAA), 100 U/mL penicillin, and 100 U/mL streptomycin. All cells were cultivated in an incubator with 90% relative humidity and 5% CO_2_ at 37 °C.

Male SD rats (6–8 weeks old) and pre-established type 2 diabetes mellitus (T2DM) model rats were purchased from Vital River Laboratory Animal Technology Co., Ltd. (Beijing, China). Animals were housed under standard conditions (22 ± 3 °C, 12 h light/dark cycle) with ad libitum access to food and water. All animal care and experimental procedures were carried out according to the guidelines and approval of Pharmaron’s Institutional Animal Care and Use Committee (IACUC) (Approval No. PK -R-06012026, date of approval 5 February 2026), and the animal experiments were designed and reported in accordance with the ARRIVE guidelines.

### 4.3. Preparation of HIP Complexes

To identify optimal counterions for semaglutide, a screening of cationic lipids was conducted. HIP complexes were prepared by mixing ionized semaglutide (dissolved in deionized water at 20 mg/mL, carrying approximately 7 net negative charges at pH 7.4) with methanolic solutions of each cationic lipid at peptide-to-lipid molar ratios from 1:2 to 1:8. The 1:8 ratio was selected as a nominal molar ratio providing a modest excess of cationic lipid. It does not represent a determined stoichiometric composition of the complex. The reaction proceeded under standardized conditions (25 ± 2 °C, 400 rpm, 2 h), after which the precipitated complexes were isolated by centrifugation (10,000 rpm for 5 min; Microfuge-30R, Beckman (Brea, CA, USA). The resulting precipitates were washed with deionized water and subsequently lyophilized to obtain the dried HIP complexes. The precipitation efficiency (PE%) was calculated using the following equation:

$$PE\left(\%\right)=\left(1-\frac{C_{Semaglutide\;in\;supernatant}}{C_{0}}\right)\times 100\%$$

***C*_Semaglutide in supernatant_** is the concentration of semaglutide in the supernatant after complexation, and *C*_0_ is the initial concentration of semaglutide before complexation. All samples were analyzed in triplicate (*n* = 3) by ultra-performance liquid chromatography (UPLC), as detailed in the Supplementary Materials.

### 4.4. Characterization of HIP Complexes

The *n*-octanol/water (log *p*) value of the SEDAB complex was first determined to evaluate its lipophilicity. Briefly, an appropriate amount of the lyophilized complex was dispersed in a 1 mL mixture of n-octanol and water (1:1, *v*/*v*), equilibrated for 24 h under continuous shaking at 25 ± 2 °C, and subsequently centrifuged at 5000 rpm for 10 min to achieve phase separation. Semaglutide concentrations in each phase were quantified using UPLC according to the method detailed in the Supplementary Materials. The log *p* value was calculated using the following equation:

$$\log P_{\text{n-octanol}/\mathrm{water}}=log\frac{c_{\text{n-octanol}}}{c_{water}}$$

*C*_n-octanol_ is the concentration of SEDAB in the n-octanol phase, and *C*_water_ is the concentration of SEDAB in the aqueous phase.

Fourier-transform infrared spectroscopy (FTIR) analysis was employed to verify charge neutralization upon complex formation (Nicolet™ iS50, Thermo Fisher, Waltham, MA, USA). Native semaglutide, native DODAB, and the SE–DODAB complex were analyzed. Samples were prepared using the potassium bromide (KBr) pellet method, and spectra were recorded over the wavenumber range of 4000–400 cm^−1^ with 16 scans per sample. Differential scanning calorimetry (DSC) was performed on native semaglutide, native DODAB, a physical mixture of semaglutide and DODAB at a molar ratio of 1:8, and the SE-DODAB complex using a DSC instrument (DSC3, Mettler Toledo, Switzerland). Samples were heated from −10 to 200 °C at a heating rate of 10 °C/min under a continuous nitrogen purge at 20 mL/min. X-ray powder diffraction (XRD) was performed on native semaglutide, native DODAB, a physical mixture of semaglutide and DODAB at a molar ratio of 1:8, and the SE-DODAB complex using an XRD instrument (D8 ADVANCE, Bruker, Karlsruhe, Germany). XRD measurements were performed at 40 kV and 40 mA using θ/2θ scanning mode over a 2θ range of 5–90° at a scanning rate of 5°/min.

### 4.5. Selection of SMEDDS Excipients Based on SEDAB Solubility

To select appropriate excipients, the saturation solubility of SEDAB was assessed in various oils, surfactants, and cosurfactants. An excess amount of the SEDAB complex was mixed with 1 mL of each excipient and shaken at 37 °C for 24 h in a thermostatic shaker (ZQZY-88C, Zhichu, Shanghai, China). After centrifugation at 10,000 rpm for 5 min, the supernatant was diluted with acetonitrile–methanol–water (2:2:1, *v*/*v*/*v*) and analyzed by UPLC to determine the drug concentration.

### 4.6. Preparation and Characterization of SEDAB-SME

Blank SME was first prepared by combining the oil phase, surfactant, and co-surfactant with stirring at 400 rpm for 10 min (25 ± 2 °C). SEDAB complex was then introduced into the blank SME, followed by ultrasonication at 37 °C for 30 min. The resulting mixture was centrifuged (10,000 rpm, 5 min) to obtain SEDAB-SME. The hydrodynamic diameter (Dh), polydispersity index (PDI), and zeta potential of the resulting SEDAB-SME nanodroplets were measured using a nanoparticle size analyzer (Nicomp Z3000, Entegris, Billerica, MA, USA). To evaluate physical stability and drug content after short-term storage, the selected SEDAB-SME preconcentrate was stored at 2–8 °C for 7 days, after which the diluted samples were re-evaluated for the aforementioned parameters.

For morphological examination, a diluted SEDAB-SME sample was deposited onto a carbon film copper mesh and gently blotted dry with filter paper. The samples were then negatively stained with phosphotungstic acid, followed by a second blotting step to remove excess stain, and dried under an infrared lamp. The microstructure was subsequently characterized using a transmission electron microscope (Talos F200X, Thermo Fisher Scientific, Waltham, MA, USA). Unless otherwise indicated, all doses and concentrations used in the cellular and animal experiments were expressed as semaglutide equivalents, calculated from the HPLC-measured semaglutide concentration of each batch.

### 4.7. Evaluation of In Vitro Proteolytic Stability of SEDAB-SME

The resistance of SEDAB-SME to proteolytic degradation was evaluated in simulated intestinal fluid (SIF, with trypsin and 0.1% porcine bile salts, Yuanye Bio-Technology Co., Ltd.) compared with native semaglutide. Samples were individually dispersed in SIF (1:2, *v*/*v*), and incubated at 37 °C under continuous shaking in a thermostatic shaker. Aliquots (300 μL) were collected at 30, 60, 90 and 120 min, immediately quenched with 4.7 mL diluent (acetonitrile/methanol/water, 2:2:1, *v*/*v*/*v*) to inactivate the enzyme, and centrifuged (10,000 rpm, 5 min). Semaglutide content in the supernatant was quantified using UPLC.

### 4.8. In Vitro Drug Release of SEDAB-SME

Drug release from SEDAB-SME was evaluated using a USP apparatus II (paddle method). The formulation and native semaglutide were each loaded into dialysis bags (MWCO 300 kDa, Solarbio Science & Technology Co., Ltd., Beijing, China) with a fixed volume of 1 mL and immersed in 500 mL of pH 6.8 phosphate buffer at 37 °C, with a paddle rotation speed of 50 rpm. Drug release from SEDAB-SME was evaluated under two conditions. In the first condition, cholesteryl esterase (0.5 U/mL) was included in the dialysis bags of both the native semaglutide and SEDAB-SME groups to disrupt the surfactant interfacial film and to facilitate the release of the encapsulated peptide under sink conditions. This condition provided an estimate of the maximum releasable drug fraction under an intestinally relevant esterase condition. In the second condition, no cholesteryl esterase was added to either group, allowing assessment of the release behavior when the interfacial film remains intact. Aliquots (1 mL) were withdrawn at predetermined time points (0.5, 1, 2, 3, 4, 5 and 6 h). Drug concentrations in the release medium were quantified by LC-MS/MS to generate the cumulative release profile.

### 4.9. In Vitro Biocompatibility Study

Caco-2 cells were seeded into 96-well plates at a density of 8 × 10^3^ cells/well and incubated for 48 h. Cells were treated with SEDAB-SME at concentrations ranging from 0.1 to 20 μg/mL (*n* = 5) for 5 h. After washing with HBSS, cells were incubated with medium containing 10% CCK-8 solution for 1 h. Absorbance was measured at 450 nm using a microplate reader (Synergy Neo2, BioTek Instruments, Winooski, VT, USA). Cell viability (%) was calculated using the following formula:

$$Cell\;viability\left(\%\right)=\frac{A_{Sample}-A_{Blank}}{A_{Control}-A_{Blank}}\times 100\%$$

*A*_Sample_ is the absorbance value of cells in the sample group; *A*_Control_ is the absorbance of cells in the control group; and *A*_Blank_ is the absorbance value of the blank wells containing medium without cells.

### 4.10. In Vitro Cellular Uptake of SEDAB-SME

The cellular uptake of SEDAB-SME was quantitatively and qualitatively analyzed by flow cytometry (FCM) and confocal laser scanning microscopy (CLSM), respectively. For this study, FITC-labeled semaglutide was prepared as follows and utilized to prepare the FITC-labeled SEDAB-SME formulation. Semaglutide (200 mg) was dissolved in 20 mL of 0.1 M carbonate buffer (pH 8.5). Under light protection and gentle stirring, FITC (1.0 equiv.) dissolved in DMF was added dropwise. The reaction mixture was wrapped in aluminum foil and stirred at room temperature for 2 h, filtered through a 0.22 µm membrane, and purified by C18 reverse-phase column chromatography using acetonitrile/water as the mobile phase. Fractions exhibiting absorbance at both 495 nm (FITC) and 220 nm (peptide) were collected, and the purity of the FITC-labeled conjugate was determined by HPLC at 495 nm based on peak-area analysis (94.66%).

For FCM analysis, Caco-2 cells were seeded into 12-well plates and cultured for 48 h and incubated with FITC-labeled SEDAB-SME or native FITC-semaglutide (10 μg/mL) for 2, 3, or 4 h. Subsequently, the cells were washed twice with HBSS and digested with 0.25% trypsin. The detached cells were then collected by centrifugation (300× *g*, 5 min) and resuspended in PBS prior to analysis. The resulting cell suspensions were analyzed using a flow cytometer (CytoFLEX LX, Beckman, Brea, CA, USA), and the mean fluorescence intensity (MFI) and the percentage of FITC-positive cells were analyzed using FlowJo software (version 10.8.1, BD Biosciences, Ashland, USA).

For CLSM observation, Caco-2 cells were seeded in confocal dishes at a density of 1 × 10^5^ cells/dish and incubated for 48 h, then treated with FITC-labeled SEDAB-SME solution (10 μg/mL) at 37 °C for 4 h. After incubation, the cells were rinsed twice with HBSS, fixed using 4% paraformaldehyde, and stained with Hoechst 33342 dye (excitation/emission = 350/460 nm) and TRITC-Phalloidin (excitation/emission = 540/565 nm), and ultimately imaged with a laser confocal microscope (AXR, Nikon, Tokyo, Japan).

### 4.11. In Vitro Study of Transepithelial Transport Efficiency

Intestinal absorption was evaluated using a Caco-2 cell monolayer model. Cells were seeded at a density of 2 × 10^5^ cells per insert in 12-well transwell plates (0.4 μm pore size, 1.12 cm^2^ growth area, Corning, NY, USA). Cells were cultured for 21 days, with the medium changed every other day for the first 7 days and daily thereafter. Monolayer integrity was monitored by measuring transepithelial electrical resistance (TEER, Millicell-ERS, Millipore, Burlington, VT, USA). Monolayers with TEER values exceeding 1000 Ω·cm^2^ were selected for subsequent transepithelial transport experiments.

For the transepithelial transport study of SEDAB-SME, the medium was removed and the cell monolayers were washed twice with HBSS. The SEDAB-SME preconcentrate was diluted 950-fold in HBSS to give the working solution, in which the final concentrations of Labrasol ALF and Kolliphor RH 40 were 0.03% (*w*/*v*) each. Then, SEDAB-SME solution (0.5 mL, 10 μg/mL) and native semaglutide solution (0.5 mL, 10 μg/mL) were applied to the apical (AP) sides, and 1.5 mL of fresh HBSS was added to the basolateral (BL) sides. TEER values were monitored at the predetermined time points throughout the experiment. After 4 h of incubation, samples were collected from both AP and BL compartments. The polycarbonate membrane with attached cell monolayer was carefully excised from the insert following three washes with cold HBSS. The semaglutide content in the samples was quantified using LC-MS/MS (as detailed in the Supplementary Materials) and the apparent permeability coefficient (*P*_app_) was calculated using the following formula:

$$P_{app}=\frac{dQ/dt}{A\times C_{0}}$$

dQ/dt represents the amount transported across the membrane per unit time, A denotes the area of each well and $C_{0}$ denotes the initial donor concentration.

### 4.12. Endocytosis and Intracellular Transport Pathways of SEDAB-SME in Caco-2 Cells

Caco-2 cells were seeded and cultured as described in Section 4.10 for both flow cytometry (FCM) and confocal laser scanning microscopy (CLSM) analyses. To investigate endocytic pathways, Caco-2 cells were pretreated with specific inhibitors methyl-β-cyclodextrin (6 mM), chlorpromazine hydrochloride (30 μM), amiloride (100 μM), or genistein (50 μg/mL) for 1 h; these concentrations were selected within the ranges reported in previous studies [42,43,44,45,46]. Subsequently, the inhibitors were removed, and FITC-labeled SEDAB-SME (10 μg/mL) in fresh medium containing the respective inhibitor was added and incubated for 4 h to maintain the inhibitory effect throughout the uptake period. To assess intracellular transport mechanisms, cells were pre-incubated with FITC-labeled SEDAB-SME (10 μg/mL) for 2 h to allow initial internalization. After washing to remove extracellular drugs, cells were further incubated for 3 h with fresh medium containing brefeldin A (25 μg/mL), monensin (32.5 μg/mL), nocodazole (6 μg/mL), or bafilomycin A1 (100 nM), as previously described [32,33,34,35].

After incubation, for FCM analysis, cells were washed twice with cold HBSS, digested with 0.25% trypsin, collected by centrifugation, resuspended in PBS, and analyzed using a flow cytometer. For CLSM analysis, the treated cells were initially rinsed with HBSS, fixed with 4% paraformaldehyde, and then stained with Hoechst 33342 dye (excitation/emission = 350/460 nm) and TRITC-Phalloidin (excitation/emission = 540/565 nm) prior to imaging.

### 4.13. In Vivo Pharmacokinetic (PK) Study

The pharmacokinetic (PK) study was performed using the intra-jejunal (i.j.) infusion method. This route was adopted as a regional intestinal absorption model for oral peptide delivery, in line with established practice in the field. Intra-jejunal administration of peptide formulations has been widely employed to quantify intestinal absorption efficiency while bypassing confounding variables such as gastric emptying, gastric proteolysis, and luminal dilution. Buckley and co-workers examined the absorption of orally delivered semaglutide co-formulated with SNAC using direct intragastric administration paired with intravenous dosing to isolate the absorption step from swallowing-related confounders [38]. Banerjee and co-workers assessed insulin-loaded choline–geranate ionic liquids by intra-jejunal administration in parallel with oral enteric-capsule administration to accurately evaluate jejunal absorption efficiency [36]. In a recent preclinical evaluation of an oral semaglutide formulation using the Axcess™ vehicle, semaglutide was instilled directly into the intestinal lumen of anesthetized rats to determine absolute bioavailability [37]. Similarly, Gedulin and colleagues characterized the pharmacokinetics of exenatide following delivery to multiple epithelial sites of the gastrointestinal tract (including intraduodenal administration) in rats, establishing a precedent for multi-site peptide absorption profiling [39]. In line with these precedents, the i.j. route here (i) delivers SEDAB-SME directly to the primary absorption site of the jejunum, (ii) bypasses gastric proteolysis, dilution, and variable gastric emptying, and (iii) provides a quantitative readout of jejunal absorption efficiency that would otherwise be masked by gastric variables. In addition, the high dose required for reliable PK quantification (7.56 mg/kg) exceeded the loading capacity of enteric-coated capsules suitable for SD rats, further supporting this methodological choice.

Prior to intra-jejunal administration, a surgical procedure was performed on SD rats. All experimental animals were fasted overnight before surgery, and all surgical instruments were sterilized via autoclaving. Thirty minutes prior to surgical incision, animals received preoperative analgesia (carprofen, 5 mg/kg, s.c.). General anesthesia was induced by intramuscular injection of zolazepam-tiletamine (7.5 mg/kg) and xylazine (1 mg/kg). A skin area of approximately 1.5 cm × 1.5 cm over the abdominal midline was shaved, disinfected with alcohol-soaked cotton balls, and wiped clean. A 1.0 cm longitudinal incision was made along the abdominal midline to expose the intestinal tissue. The jejunum was accurately identified, and a custom cannula (0.02 inches inner diameter, 0.037 inches outer diameter) was inserted 10–15 mm proximal to the distal end of the jejunum with the assistance of a 22G needle. The cannula was advanced slowly until the first silicone bead (1 cm away from the cannula tip) anchored at the intestinal puncture site. Subsequently, at the catheter puncture site, sutures were passed through the intestinal wall for a length of approximately 1 mm, tied, and the suture ends were then tied over the catheter to secure it. The abdominal musculature was closed with absorbable suture; the abdominal skin incision was closed with non-absorbable suture. The subscapular exteriorization site was closed with stainless steel wound clips. Postoperatively, all animals were placed on a constant-temperature warming pad until they fully recovered from anesthesia. All animals were allowed to fully recover for at least 96 h before subsequent formal experimental procedures.

Male Sprague Dawley (SD) rats (6–8 weeks) were randomly assigned to three groups (*n* = 5). Animals were randomized by body-weight ranking: rats were ranked by body weight and allocated across the treatment groups in rank order, so that body-weight distributions were balanced among groups. The allocation information and treatment codes were known to the investigator responsible for treatment administration. The investigator responsible for sample quantification was blinded to treatment allocation during data acquisition and quantification, while the group-allocation information was maintained separately by the first investigator. Prior to the experiment, all rats were fasted overnight (12–16 h) with free access to water. The treatments were administered as follows: subcutaneous semaglutide (1.0 mg/kg); intra-jejunal semaglutide (7.56 mg/kg); intra-jejunal SEDAB-SME (7.56 mg/kg). The i.j. formulations were administered through the indwelling cannula over approximately 120 s: native semaglutide, prepared in PBS at a final concentration of 2.5 mg/mL, was administered as 0.756 mL per rat (3.02 mL/kg); SEDAB-SME, prepared at 9 mg/mL and diluted 1:3 (*v*/*v*) in PBS (final semaglutide concentration 2.25 mg/mL), was administered as 0.84 mL per rat (3.36 mL/kg); both corresponded to the 7.56 mg/kg dose stated above. Blood samples (200 μL) were collected from the jugular vein at time points (5 min, 10 min, 30 min, 45 min, 1 h, 2 h, 4 h, 6 h, 8 h, 12 h, 24 h and 48 h) in centrifuge tubes pre-filled with EDTA-K2. Samples were kept on ice before being centrifuged (4000 g, 5 min, 4 °C) and were stored in a freezer at −80 ± 5 °C prior to analysis. Plasma semaglutide concentrations were determined by a validated LC-MS/MS method (Supplementary Materials). The relative bioavailability (Frel%) was calculated using the following formula:

$$Frel\%=\frac{{AUC}_{test}\times {Dose}_{s.c.}}{{AUC}_{s.c.}\times {Dose}_{test}}\times 100\%$$

where *AUC*_test_ and *Dose*_test_ are the *AUC*_0−48_ and the administered dose of the respective intra-jejunal formulation (native semaglutide or SEDAB-SME, 7.56 mg/kg), and *AUC*_s.c._ and *Dose*_s.c._ are those of subcutaneous semaglutide, the reference route (1.0 mg/kg). Frel therefore expresses the dose-normalized systemic exposure of each i.j. formulation relative to subcutaneous injection within this regional intestinal absorption model [36]; it does not represent oral bioavailability.

### 4.14. In Vivo Glucoregulatory Efficacy of the SEDAB-SME

For the pharmacodynamic study, pre-established T2DM model rats were used, in which diabetes had been induced by the vendor via 4 weeks of high-fat and high-sugar diet (HFHSD) feeding followed by a single intraperitoneal injection of streptozotocin (STZ, 35 mg/kg) after an overnight fast [60]. Stable hyperglycemia was confirmed before release, with fasting blood glucose (FBG) levels exceeding 11.1 mmol/L measured on days 3 and 7 post-STZ injection. Prior to the experiment, all rats were fasted overnight (12–16 h) with free access to water. This low-dose STZ regimen combined with prior HFHSD feeding is an established T2DM model that combines diet-induced insulin resistance with partial beta-cell dysfunction while preserving residual beta-cell function, which is required for the glucose-dependent insulinotropic action of GLP-1 receptor agonists; high-dose STZ protocols (60–100 mg/kg), by contrast, induce near-complete beta-cell ablation and model type 1 diabetes [60]. This model has likewise been used to demonstrate the acute oral efficacy of semaglutide formulations in rats [50], and its limitations, including inter-animal variability in the severity of hyperglycemia, are acknowledged.

In the single-dose study, T2DM rats were randomly divided into four groups (*n* = 5): (1) SEDAB-SME enteric-coated capsule (2 mg/kg semaglutide, i.g.); (2) semaglutide solution (2 mg/kg semaglutide, i.g.); (3) subcutaneous (s.c.) semaglutide solution (0.02 mg/kg); and (4) T2DM model control (oral saline, i.g.). Randomization and blinding were performed as described in Section 4.13. Prior to dosing, fasting blood glucose was measured for each animal, and the 0 h value was used as the individual baseline. Blood glucose levels during the 6-h monitoring period are expressed as percentages of this baseline to account for inter-animal variability in absolute glucose values. Three enteric-coated capsules of SEDAB-SME (YG01-2, coating material: hypromellose phthalate, length when locked maximum: 5.0 mm, capsule body capacity: ~25 μL, Yuyan Instruments, Shanghai) were orally administered to rats using a specialized capsule gavage needle (Yuyan Instruments, Shanghai, China).

The 6-h monitoring window was selected based on the markedly shorter terminal half-life of semaglutide in rats (~7–12 h) compared with that in humans (~160 h) [61,62], in line with the established use of acute, hour-level glucose monitoring with tail-tip blood sampling and AUC analysis for evaluating the pharmacodynamic activity of GLP-1-related peptide (oxyntomodulin) formulations [63]. To better reflect the clinical use scenario of GLP-1 receptor agonists, where patients typically fast overnight before dosing but resume food intake within hours, food was restored 2 h post-administration. Given that the primary clinical benefit of GLP-1 receptor agonists lies in suppressing postprandial glucose excursions [49], this two-phase design allows simultaneous evaluation of fasting-phase glucose lowering (0–2 h) and postprandial glucose control (2–6 h). Blood glucose levels were monitored at 0, 1, 2, 3, 4, 5 and 6 h post-dosing via tail tip bleeding using a blood glucose meter (Accu-Chek, Roche, Basel, Switzerland). The *AUC*_0–6h_ was calculated from the absolute blood glucose values using the linear trapezoidal method; blood glucose levels in figures are expressed as percentages of the pre-dose (0 h) baseline for visualization.

### 4.15. Statistical Analysis

All data were presented as mean ± standard deviation (SD). Statistical analyses were performed using GraphPad Prism (V.10.6.0, GraphPad Software, Boston, MA, USA). Unpaired two-tailed Student’s *t*-test was employed for two-group comparisons, while one-way analysis of variance (ANOVA) followed by Dunnett’s or Tukey’s multiple-comparisons test was used for multiple-group comparisons. For experiments involving both treatment and time as independent factors, a two-way ANOVA was performed. The blood-glucose time course involved repeated measurements of the same animals and was therefore analyzed by a two-way repeated-measures ANOVA. In this model, group was treated as a between-subjects factor, time as a within-subjects factor, and the group × time interaction was tested. Sphericity was not assumed, and the Geisser–Greenhouse correction was applied. When a significant group × time interaction was detected, all pairwise between-group comparisons at each time point were performed using Tukey’s multiple-comparisons procedure. Comparisons within each time point were treated as a separate family, and multiplicity-adjusted *p* values are reported. The *AUC*_0−6h_ values were compared by one-way ANOVA followed by Tukey’s test. The specific statistical test applied to each experiment is indicated in the corresponding figure legend. A statistically significant difference was considered when the *p* value < 0.05.

## Figures and Tables

**Table 1:** Solubility of the SEDAB complex in various pharmaceutical excipients.

Function	Material	Solubility (mg/mL)
Oil	Labrafac MC60	6.5 ± 1.9
	Peceol^®^	0.00180 ± 0.00023
	MCT	0.00051 ± 0.00047
	Capryol^®^ 90	0.00036 ± 0.00012
Surfactant	Labrasol ALF	0.790 ± 0.030
	Tween 80	0.300 ± 0.020
	Kolliphor RH 40	0.280 ± 0.060
	Kolliphor^®^ HS 15	0.049 ± 0.020
Co-surfactant	Propylene glycol	1.95 ± 0.11
	Transcutol^®^ P	2.44 ± 0.88
	PEG-400	0.26 ± 0.10
	Ethylene glycol	6.94 ± 0.26

**Table 2:** Pharmacokinetic parameters of semaglutide formulations in SD rats (*n* = 5).

Formulations	Semaglutide (s.c.)	Semaglutide (i.j.)	SEDAB-SME (i.j.)
Dose (mg/kg)	1.0	7.56	7.56
*t*_1/2_ (h)	15.6 ± 2.3	7.4 ± 3.9 *	6.34 ± 0.77
*T*_max_ (h) ‡	8.0 (6.0–12.0)	0.167 (0.083–2.0)	0.167 (0.167–0.50)
*C*_max_ (ng/mL)	(2.20 ± 0.18) × 10^3^	4.1 ± 4.9 *	147 ± 55
*AUC*_0–48_ (ng.h/mL) †	(7.16 ± 0.98) × 10^4^	23 ± 23 *	(6.0 ± 3.0) × 10^2^
*AUC*_0–∞_ (ng * h/mL)	(8.2 ± 1.3) × 10^4^	(5.4 ± 3.9) × 10^1^	(6.7 ± 3.2) × 10^2^
%*AUC* extrapolated (%)	12.3 ± 3.3	4.6 ± 2.5 × 10^1^	5.3 ± 1.4
*F*_rel_ (%)	100.00	0.004	0.12

* The t_1/2_, Cmax, and *AUC*_0–48_ of i.j. semaglutide were estimated from plasma concentrations near the lower limit of quantification (LLOQ) and should be interpreted with caution. † *AUC*_0−48_ was calculated from time zero to 48 h using the linear trapezoidal method. ‡ *T*max is reported as median (range) owing to its non-parametric distribution. All reported pharmacokinetic (PK) parameters were calculated by non-compartmental analysis (NCA) using Phoenix WinNonlin (Version 8.3.5, Certara, Princeton, USA), Cmax, *AUC*_0–48_ and AUC_0–∞_ were expressed as arithmetic mean ± SD. Frel (%) is the dose-normalized relative bioavailability versus subcutaneous semaglutide, calculated as (*AUC*_0−48,test_ × *Dose*_s.c._)/(*AUC*_0−48,s.c._ × *Dose*_test_) × 100% (Section 4.13); it is not oral bioavailability.

## Data Availability

The data that support the findings of this study are available from the corresponding author upon reasonable request.

## Supplementary Materials

The following supporting information can be downloaded at: https://www.mdpi.com/article/10.3390/ijms27219523/s1.

## Abbreviations

The following abbreviations are used in this manuscript:

T2DM	type 2 diabetes mellitus
GLP-1	glucagon-like peptide-1
SMEDDS	self-microemulsifying drug delivery system
HIP	hydrophobic ion pairing
DODAB	dioctadecyldimethylammonium bromide
TOAB	tetraoctylammonium bromide
MEM	Minimum Essential Medium
FBS	fetal bovine serum
HPLC	high-performance liquid chromatography
LC-MS/MS	liquid chromatography–tandem mass spectrometry
FTIR	Fourier-transform infrared spectroscopy
DSC	differential scanning calorimetry
XRD	X-ray powder diffraction
TEER	transepithelial electrical resistance
Papp	apparent permeability coefficient
TEM	transmission electron microscopy
Dh	hydrodynamic diameter
PDI	polydispersity index
SIF	simulated intestinal fluid
HFHSD	high-fat and high-sugar diet
STZ	streptozotocin
CCK-8	Cell Counting Kit-8
MβCD	methyl-β-cyclodextrin
CPZ	chlorpromazine
FCM	flow cytometry
CLSM	confocal laser scanning microscopy
FITC	fluorescein isothiocyanate
MFI	mean fluorescence intensity
PK	pharmacokinetic
SD rats	Sprague Dawley rats
i.g.	Intragastric administration
i.j.	Intra-jejunal
s.c.	subcutaneous
FBG	fasting blood glucose
AUC	area under the curve
Cmax	maximum plasma concentration
Tmax	time to reach maximum plasma concentration
Frel	relative bioavailability
