# Supplementary material for: A Hydrophobic Ion-Paired Self-Microemulsifying System Enhances Jejunal Absorption and Acute Glycemic Control of Semaglutide in Rats
Source: Int J Mol Sci. 2026 Oct 24;27(21):9523. doi: 10.3390/ijms27219523 (PMC13762768; doi:10.3390/ijms27219523)
Supplement: Supplementary file 1 [file ijms-27-09523-s001.zip › ijms-4614374-supplementary.pdf]

Supplementary Materials

A hydrophobic ion-paired self-microemulsifying system enhances jejunal absorption and acute glycemic control of semaglutide in rats

Ke Wang [1,†], Yijie Hu [1,†], Yicheng Jiang [1], Ruixin Fan [1], Yuzhang Bao [1], Sikai Wang [1], Qian Liang [1], Kaili Lu [1], Junjie Xue [1], Yi Yan [2], Xiaoyan Liu [1,2], Wanhui Liu [1], Yuanjun Zhu [1,2,*] and Jiancheng Wang [1,2,*]

[1] Laboratory of Innovative Formulations and Pharmaceutical Excipients, Peking University Ningbo Institute of Marine Medicine, Ningbo 315832, China;

[2] Beijing Key Laboratory of Molecular Pharmaceutics and New Drug Delivery Systems, State Key Laboratory of Natural and Biomimetic Drugs, School of Pharmaceutical Sciences, Peking University, Beijing 100191, China;

* Correspondence: zhuyuanjun@bjmu.edu.cn (Y.Z.); wang-jc@bjmu.edu.cn (J.W.)
† These authors contributed equally to this work.

Quantification of Semaglutide by UPLC and LC-MS/MS

A UPLC method was developed and validated for the quantification of semaglutide in drug loading and *in vitro* release studies. Analyses were performed on an ACQUITY Premier UPLC system (Waters, USA) equipped with a Quaternary Solvent Manager, a Sample Manager FTN, an ACQUITY UPLC 30 cm Column Heater (CH-30A), and a PDA eλ detector. The chromatographic conditions were summarized in Table S2.

The liquid chromatography-tandem mass spectrometry (LC-MS/MS) was employed for the quantification of semaglutide in cell samples and plasma samples. *For cell samples:* 20 μL aliquots of the apical solution and basolateral solution were each mixed with 100 μL of internal standard solution. The mixtures were vortexed for 10 min and then centrifuged at 12,000 rpm for 10 min at 4°C, and the supernatants were subsequently collected. *For plasma samples:* a 60 μL aliquot of sample (30 μL plasma and 30 μL blank male Sprague–Dawley rat plasma) was mixed with 180 μL of methanol containing the internal standard (50 ng/mL) to precipitate proteins. The mixture was vortexed for 10 min and then centrifuged at 3,900 rpm for 15 min at 4°C. The resulting

supernatant was diluted 1.5-fold with water, and a 10 μL aliquot was injected into the LC–MS/MS system. Chromatographic separation was achieved on a Waters Acquity Premier CSH C18 1.7μm VanGuard FIT 2.1*100 mm. The mobile phase consisted of 0.1% formic acid in deionized water (A) and 0.1% formic acid in acetonitrile (B), with a gradient elution program as detailed in Table S3 and the mass parameters for the detection of semaglutide were summarized in Table S4.

Table S1. Cationic lipids screened for hydrophobic ion pairing with semaglutide.

Cationic lipid	Chemical structure
DODAB	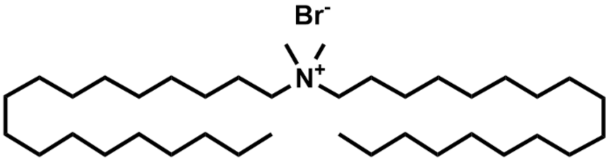
TOAB	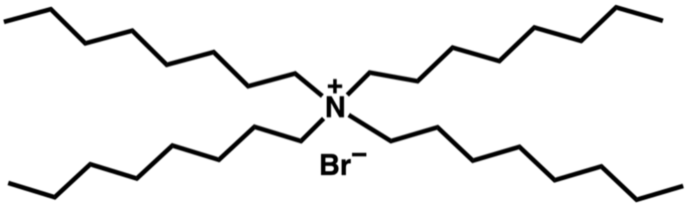

Table S2. Chromatographic conditions for the UPLC method.

Parameter	Condition
Stationary phase	ACQUITY^{TM} Premier Peptide BEH C18 2.1 x100 mm,1.7 µm
Duration	22 min
Column temperature	42 °C
Autosampler temperature	25 °C
Mobile phase	A: 80 mM$(NH_4)_2HPO_4$ buffer (pH 3.6, adjusted with 85% phosphoric acid)–ACN–H_2O (8:8:1, v/v/v)<br>B: ACN–IPA–H_2O (3:3:1, v/v/v)
Injection volume	1 µL
Flow rate(mL/min)	0.4 mL/min
Gradient	0-17 min: 48% B; 17-17.1 min: 48→100% B; 17.1-19.5 min: 100% B; 19.5-19.8 min: 100→48% B; 19.8-22 min: 48% B
Detection	215 nm

Table S3. UPLC conditions for the LC-MS/MS method

Parameter	Condition
Stationary phase	Acquity Premier CSH C18 1.7μm VanGuard FIT 2.1*100 mm
Duration	6 min
Column temperature	60 °C
Autosampler temperature	7 °C
Mobile phase	A: 0.1% formic acid in water<br>B: 0.1% formic acid in acetonitrile
Injection volume	10 µL
Flow rate(mL/min)	0.3 mL/min
Gradient	0-0.2 min: 35% B; 0.2-3.2 min: 35→45% B; 3.2-3.3 min: 45→90% B; 3.3-4.5 min: 90% B; 4.5-4.6 min: 90→35% B; 4.6-6.0 min: 35% B

Table S4. MS parameters for the detection of semaglutide (with liraglutide as internal standard)

Parameter	Condition
Ionization mode	ESI^+
Scan mode	MRM
Desolvation gas flow	1000 L/h
Desolvation temperature	550 °C
Capillary voltage	2 kV
Cone voltage	30 V
MS acquisition window	1.5–6.0 min

Compound	Precursor ion (m/z)	Product ion (m/z)	Collision energy (eV)
Semaglutide	1029.3	1238.1	32
Liraglutide (IS)	938.7	1128.5	32

Table S5. Experimentally determined semaglutide concentrations in independently prepared batches of SEDAB-SME.

SEDAB-SME batch	Semaglutide concentration (mg/mL)	Mean (mg/mL)
**Batch 1**	9.62<br>9.46<br>9.50	9.53

**Batch 2**	9.04<br>9.03	9.03
**Batch 3**	8.22<br>8.11	8.17
**Batch 4**	8.72<br>8.84	8.78

Table S6. Changes in semaglutide content in the self-microemulsion after 7 days of storage at 2–8 °C (n = 3).

Time (d)	0 d	2–8 °C stored for 7 d	7 d / 0 d
Assay (mg/mL)	8.21 ± 0.15	6.74 ± 0.29	82.1%

Note: The data show a mean semaglutide content retention of 82.1% after 7 days at 2–8 °C, indicating an approximately 18% loss. The underlying cause (e.g., adsorption, precipitation, or degradation) was not determined in this study. These data do not establish chemical stability.

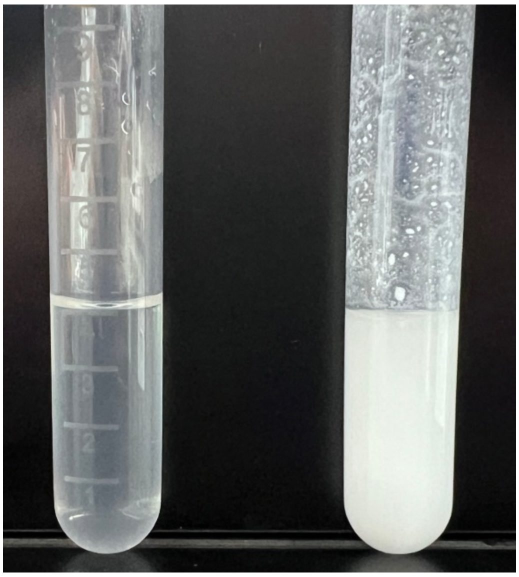

Figure S1. Visual appearance of semaglutide (left) and semaglutide–DODAB HIP complex (right) after preparation under identical conditions. Semaglutide alone remained clear, whereas the complex formed a visible precipitate. Supernatant analysis showed that semaglutide was almost undetectable after complexation, consistent with its incorporation into the precipitated phase.
